# Identification keys to the *Anopheles* mosquitoes of South America (Diptera: Culicidae). II. Fourth-instar larvae

**DOI:** 10.1186/s13071-020-04299-5

**Published:** 2020-11-18

**Authors:** Maria Anice Mureb Sallum, Ranulfo González Obando, Nancy Carrejo, Richard C. Wilkerson

**Affiliations:** 1 Departamento de Epidemiologia, Faculdade de Saúde Pública, Universidade de São Paulo, Avenida Doutor Arnaldo 715, São Paulo, São Paulo CEP01246-904 Brazil; 2 Departamento de Biología, Universidad del Valle, A.A 25360 Cali, Colombia; 3 Department of Entomology, Smithsonian Institution, National Museum of Natural History (NMNH), Washington, DC 20560 USA; 4 Walter Reed Biosystematics Unit, Smithsonian Institution Museum Support Center, 4210 Silver Hill Rd., Suitland, MD 20746 USA; 5 Walter Reed Army Institute of Research, 503 Robert Grant Avenue, Silver Spring, MD 20910 USA

**Keywords:** *Anopheles*, Morphology, Illustrations, Identification key, Fourth-instar larvae

## Abstract

**Background:**

Accurate species identification of South American anophelines using morphological characters of the fourth-instar larva is problematic, because of the lack of up-to-date identification keys. In addition, taxonomic studies, employing scanning electron microscopy of the eggs and DNA sequence data, have uncovered multiple complexes of morphologically similar species, and resulted in the resurrection of other species from synonymy, mainly in the subgenus *Nyssorhynchus*. Consequently, the identification keys urgently need to be updated to provide accurate morphological tools to identify fourth-instar larvae of all valid species and species complexes.

**Methods:**

Morphological characters of the fourth-instar larvae of South American species of the genus *Anopheles* were examined and employed to elaborate a fully illustrated identification key. For species for which no specimens were available, illustrations were based on published literature records.

**Results:**

A fully illustrated key to the fourth-instar larvae of South American species of the genus *Anopheles* (Diptera: Culicidae) is presented. Definitions of the morphological terms used in the key are provided and illustrated.

**Conclusions:**

Morphological identification of South American *Anopheles* species based on the fourth-instar larvae has been updated. Characters of the spiracular apparatus were determined useful for the identification of morphologically similar species, in the Strodei Group and some taxa in the Myzorhynchella Section. The single *versus* branched abdominal seta 6-IV used to differentiate Myzorhynchella species from other *Nyssorhynchus* species was shown to be variable in Myzorhynchella species. Also, the abdominal setae 1-IV,V of *Anopheles atacamensis* and *Anopheles pictipennis* were shown to be slightly serrate at the edges. Recognition of this character is important to avoid inaccurate identification of these species as members of the subgenus *Anopheles*.
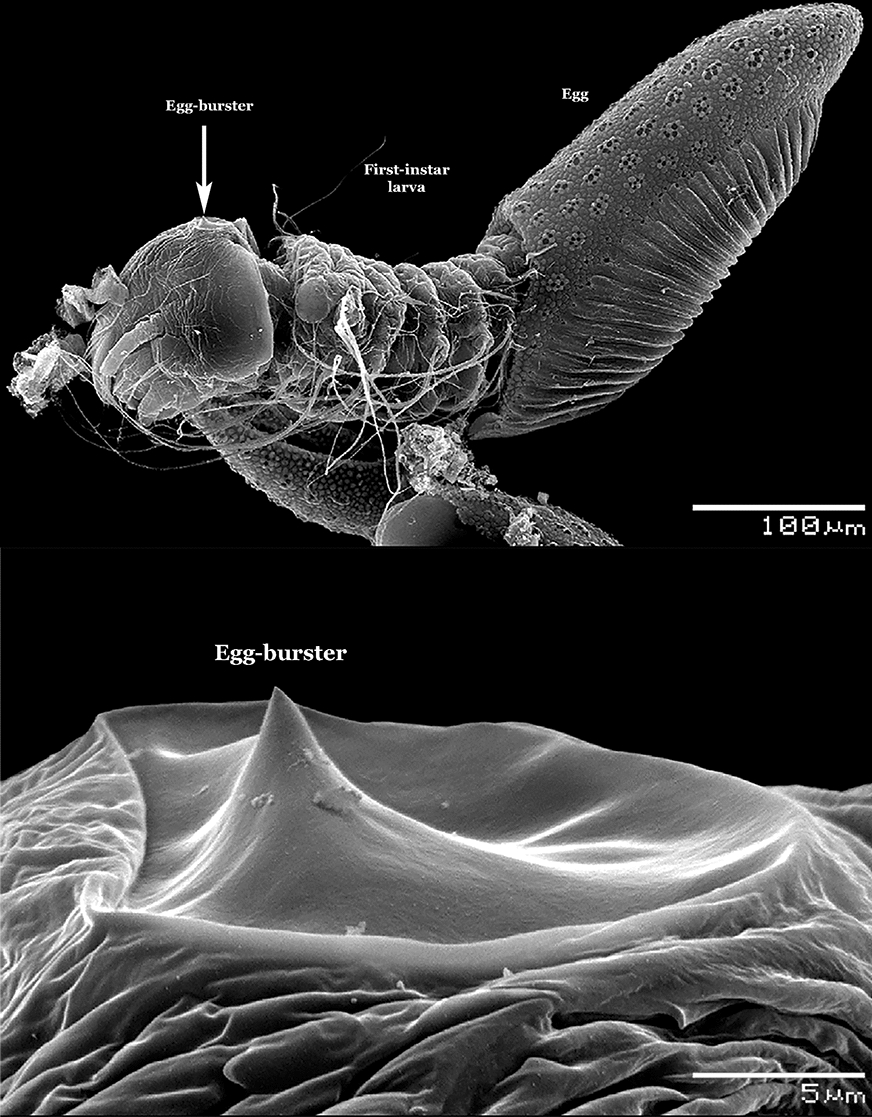

## Background

General introductory comments, distributions and species authors and publication dates are given in Part I [1] of this series of four articles. Despite the continuous interventions to control and eliminate malaria, this disease remains an important cause of morbidity and mortality in endemic tropical and subtropical countries throughout the world. Malaria occurrence is affected by several factors including those of the parasites, human host, anopheline mosquitoes and environment [2]. Because of its intrinsic complexity, in order to reach an effective and sustainable control of malaria it is necessary to adopt an integrated approach delineated on the evidence of local characteristics of the disease and transmission dynamics [3]. Thus, the public health policy for malaria control demands interventions focused on the local anopheline vector species and empirical field-knowledge on the characteristics of the local populations behavior and ecology [4]. Different mosquito species exhibit distinct preferences for blood-feeding on animals or humans, place where females take their blood-feeding, peak biting time, resting places and habitats. These biological characteristics of vector species need to be considered for the choice of an effective vector control intervention. It is important to note that vector control interventions need to be delineated for the local vector species, thus an accurate species identification is of vital importance for control, surveillance, and field malariology studies. Misidentification will cause error for a decision-making control programme that is based on metrics of transmission and local vector species [3]. Actions for vector control encompasses activities focused on the larval habitat to prevent the production of adult mosquitoes. This requires specific measures that are defined taken into consideration field-collected data of the vector population, and accurate identification of species and their habitats. The fastest, easiest, and cheapest way to identify field-collected mosquitoes for surveillance and control programmes is based on morphological characteristics. This activity is usually conducted by field-entomologists conducting mosquito collections, dissections of females in malaria endemic areas. A dichotomous morphological key is proposed with the purpose of providing a tool for identification of *Anopheles* species in South America based on the morphology of the fourth-instar larvae. The key includes major vectors, local vectors and species that have not been involved in the malaria transmission but can emerge and become dominant in human-dominated environments.

## Methods

Morphological characters of the fourth-instar larvae of South American species of the genus *Anopheles* Meigen were examined and employed to construct a fully illustrated identification key. The primary types (holotypes and paratypes) and other field-collected specimens deposited in the Coleção Entomológica de Referência, Faculdade de Saúde Pública, Universidade de São Paulo, São Paulo, Brazil (FSP-USP), Museo de Entomología, Universidad del Valle, Santiago de Cali, Colombia (MUSENUV) and the US National Mosquito Collection, Smithsonian Institution, Washington, DC, USA (USNMC) were examined to discover characters to be used in the key based on larval morphology. For species that we could not access, drawings were based on published illustrations. Photomicrographs of relevant characters for the key were taken from the fourth-instar larval exuviae mounted on microscope slides and covered with a coverslip. Specimens were obtained either as field-collected larvae or progenies of field-collected females linked to either adults. The identification was based on the morphology of the male genitalia and females. Photomicrographs were taken with a digital Canon Eos T3i (Canon, USA) camera, attached to a Leitz Diaplan microscope, using the Helicon Focus software (https://www.heliconsoft.com/heliconsoft-products/helicon-focus/) that was used to build single in-focus images by stacking multiple images of the same structure. These in-focus images were, then, employed to draw the line illustrations of the characters in CorelDRAW software (https://www.coreldraw.com/en/product/coreldraw/essentials/?topNav=en). Except for one figure, illustrations are not to scale, but the proportions of the characters in the drawings are maintained. The morphological terminology employed in the key are defined and illustrated in accordance with Harbach & Knight [5, 6]. The key is modified after Faran & Linthicum [7] and Forattini [8], with additional characters proposed herein. The species included in this Part II are listed in Table 1 of Sallum et al. [1] except for *An. acanthotorynus* Komp, 1937, *An*. *albertoi* Unti, 1941, *An*. *arthuri* Unti, 1941, *An*. *bustamantei* Galvão, 1955, *An*. *canorii* Flock & Abonnenc, 1945, *An*. *evandroi* da Costa Lima, 1937, *An*. *nigritarsis* (Chagas, 1907), *An*. *pseudomaculipes* (Chagas in Peryassú, 1908), *An*. *pseudopunctipennis levicastilloi* Leví Castillo, 1944, *An*. *pseudopunctipennis neghmei* Mann, 1950, *An*. *pseudopunctipennis noei* Mann, 1950, *An*. *pseudopunctipennis patersoni* Alvarado & Heredia, 1947, *An*. *pseudopunctipennis rivadeneirai* Leví Castillo, 1945, *An*. *rachoui* Galvão, 1952, *An*. *sanctielii* Senevet & Abonnenc, 1938 and *An*. *striatus* SantʼAna & Sallum, 2016.

## Results and discussion

### Glossary of morphological terms

**Fig. 1:**
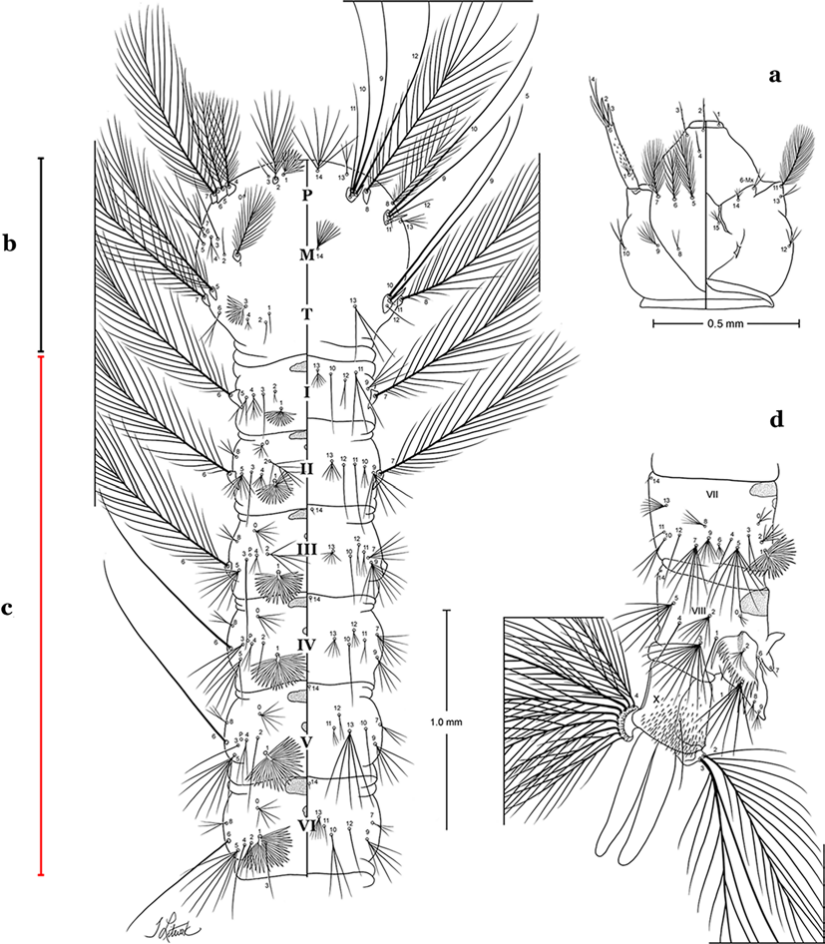
Fourth-instar larva of *An. goeldii* Rozeboom & Gabaldon, 1941. **a** Head, dorsal aspect, left, ventral aspect, right. **b** Thorax, dorsal aspect, left, ventral aspect, right. **c** Abdominal segments I–VI, dorsal aspect, left, ventral aspect, right. **d** VII–X, lateral aspect

All mosquitoes pass through four larval instars (stadia, stages). As in all insects, mosquitoes have three body regions. These are well differentiated in mosquito larvae: the head (C), thorax (T) and abdomen (A) (Fig. 1). Mosquito larvae are metapneustic, meaning there is a single pair of respiratory openings caudally. Species of the subfamily Anophelinae Grassi, 1900, which includes the genus *Anopheles* Meigen, 1818, differ from the other culicid subfamily, Culicinae Meigen, 1818, in that the larval stages do not possess a respiratory siphon, but instead have paired spiracular openings on a clearly differentiated plate.

**Fig. 2:**
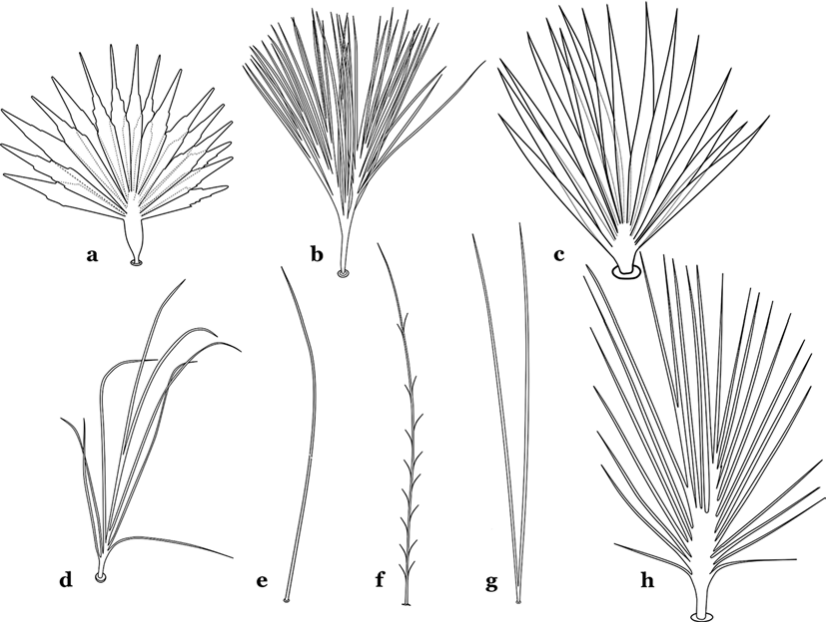
Types of common setae on larval *Anopheles*. **a** Palmate with serrate margins. **b** Dendritic. **c** Palmate with smooth margins. **d** Branched seta with filamentous branches. **e** Simple. **f** Aciculate. **g** Bifurcate. **h** Plumose

The larval cuticle (exoskeleton) bears a number of features of taxonomic utility. The most distinctive of these are the numbered, serially homologous (comparable between segments) setae that can almost always be directly compared to all other mosquito species. The setae can vary in number, form, and position (also referred to as chaetotaxy). Non-segmental structures, such as antennae and mouthparts, also have homologous numbered setae. Since setae are added with each of the four instars, the numbering system is based on the last (4th) instar. Not all setae have taxonomic value. Therefore, we only discuss those that appear in our key, or may appear in other commonly used keys. Larvae usually possess 222 pairs of setae [5], 54 on the head (including antennae and mouthparts), 42 on the thorax and 126 on the abdomen. The primary types of setae, i.e. palmate, dendritic, branched, simple, aciculate, bifurcate, and plumose are illustrated (Fig. 2). The setae are designated with a number and a structure/segment abbreviation, e.g. 2-C is seta 2 on the head, and 1-A is seta 1 on the antenna. Any given seta on a segment is expressed in the singular even though there are two with the same number per segment, e.g. seta 2-C in the above example is expressed with a singular verb, i.e. “seta 2-C is….” not “setae 2-C are….”. Singular is also used for the same numbered seta on different segments, e.g. seta 1 on abdominal segments II through VII would be “seta 1-II–VII is….”. For different numbered setae on the same or multiple segment(s) the plural is used, e.g. setae 3 and 4 on abdominal segments II and III would be “setae 3,4-II, III are….”. On any given structure/segment the setae are numbered in ascending order beginning anteriorly or from the dorsomesal line. Species of the genus *Anopheles* can be differentiated from species of the genera *Chagasia* Cruz, 1906 and *Bironella* Theobald, 1905, also the Anophelinae, by characters of the spiracular apparatus. In *Chagasia*, the posterolateral spiracular lobes possess a fringe of setae laterally, and the anterior spiracular lobe is produced into an elongate process. Species of *Anopheles* and *Bironella* lack the fringe of setae and the anterior spiracular lobe is knob-like (see [9]). Note however that *Bironella* spp. occur in the Australasian Region, including Papua New Guinea, Solomon Islands, Bismarck and Queensland, Australia.

#### Head

**Fig. 3:**
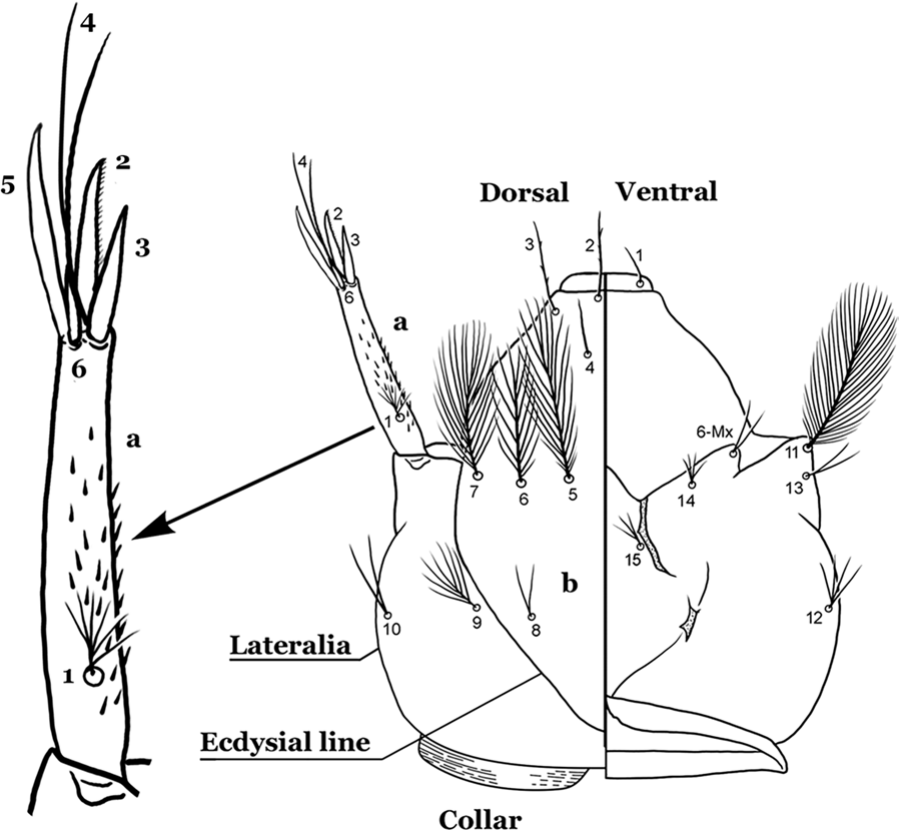
Head of fourth-instar larva of *An. goeldii*. **a** Antenna. **b** Dorsal apotome

The head is the most heavily sclerotized part of the body. It is usually longer than wide and, as in other culicids, is made up of various named sclerites (sclerotized plates). The head articulates with the thorax in such a way that is possible for larvae to rotate their heads up to 180°, resulting in great mobility while feeding. The head exhibits well-developed mandibles, maxillae, maxillary palpi and associated structures (for details see [5, 6]). These are not utilized here but can be seen elsewhere in the literature. Anterolaterally there is a pair of antennae (A), which are made up of a very short scape and a long tubular structure formed by fusion of the antennal pedicel and flagellum. All insects have some variation of these three parts of the antenna (scape, pedicel and flagellum). On the antenna there are six pairs of setae, some of which are often called by informal names: antennal seta (1-A), a terminal antennal seta (4-A) and two setae in the form of a sabre (2-A and 3-A), one dorsal and the other ventral (Fig. 3). Seta 1-A is often used in identification since its branching and position can vary. Seta 4-A can be two- or three-branched, with symmetrical or asymmetrical branching.

The head has 15 pairs of setae, a number of which have taxonomic utility. These setae are often referred to by informal names. Head setae 2-C, 3-C and 4-C are the clypeal setae. Setae 2-C and 3-C are located toward the most anterior part of the head and are called the anterior clypeal setae, with the lateral seta (3-C) called external clypeal and the more mesal seta called the inner clypeal. These setae can be simple, aciculate, barbed, plumose, branched with simple branches, or dendritic, and can vary in length. The two seta 2-C can be variably separated and are considered close when the distance between them is less than the distance between setae 2-C and 3-C on either side. If the distance between the two setae 2-C is equal or more than the distance between 2-C and 3-C they are considered well separated. The ratio between the distances between 2-C and 3-C on one of the sides with relation to the distance between the bases of the pair of 2-C, constitutes the clypeal index (distance between 2-C and 3-C on one side / distance separating setae 2-C [7]). Posterior to the anterior clypeal setae is the posterior clypeal seta (4-C), which can be single, bifurcate, forked, or multi-branched, short or long. Length is judged by how far forward the seta extends toward the bases of setae 2,3-C, and development compared to development of setae 2,3-C. Medially on the head are three pairs of setae often called the frontal setae: 5-C, 6-C and 7-C (5–7-C) (Fig. 3). They are generally long and extend at least past seta 4-C. They can be simple or branched, and sometimes appear simple but are slightly branched only apically. They usually occur in a line, but 7-C can be more anterior. Seta 8-C is posterolateral to 5–7-C. Setae 9–14-C are on an area called the lateralia (lat) (lateral and ventral areas of the head lateral to the ecdysial lines (Fig. 3). Of these, 9-C is located immediately lateral of the frontal ecdysial line and near 8-C, while 10-C is lateral of 9-C. Seta 11-C can also have taxonomic utility for the diagnosis of some species, and is found dorsolaterally near the base of the antenna.

#### Thorax

**Fig. 4:**
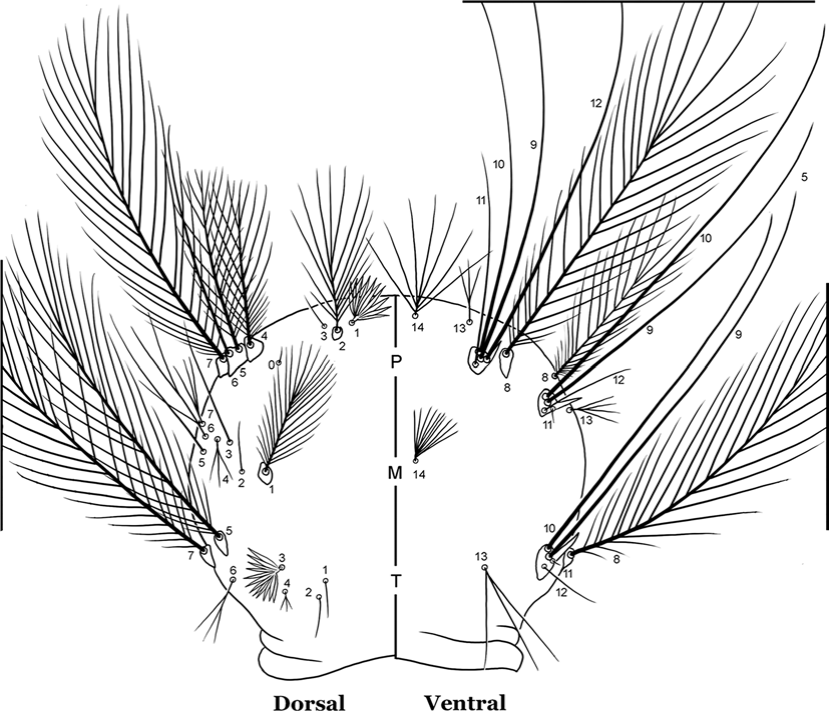
Thorax of *An. goeldii*, dorsal aspect, left; ventral aspect, right. P, prothorax; M, mesothorax; T, metathorax

The thorax is longer than wide and, as in all insects, is composed of three segments. In mosquito larvae the segments are not clearly differentiated. Using known setal groups as landmarks, one can recognize them even without distinct demarcations. They are the prothorax (P) (nearest the head), the mesothorax (M) and the metathorax (T) (Fig. 4). There are 42 pairs of setae on the thorax, many with informal names. Near the midline on the prothorax are setae 1–3-P, the submedian prothoracic group. These are often referred to their relative positions: internal (1-P), median (2-P) and external (3-P). Seta 1-P originates closest to the dorsomesal line and is generally branched or palmate. It is close to seta 2-P. Seta 3-P is usually simple and originates close to 1,2-P, which can share the same basal support plate or occur individually. These three setae are of great taxonomic utility. On the mesothorax and metathorax setae 1 and 2 (1-M,T and 2-M,T) can be closer to each other and well separated from seta 3. The variable form of seta 3-T makes it useful in the identification of some species. Setae 9–12-P,M,T, the pro-, meso- and metathoracic pleural groups, originate on a common tubercle in a lateroventral position on all three segments. Some of the setae vary in form, size, and number of branches, which often makes them taxonomically useful.

#### Abdomen

**Fig. 5:**
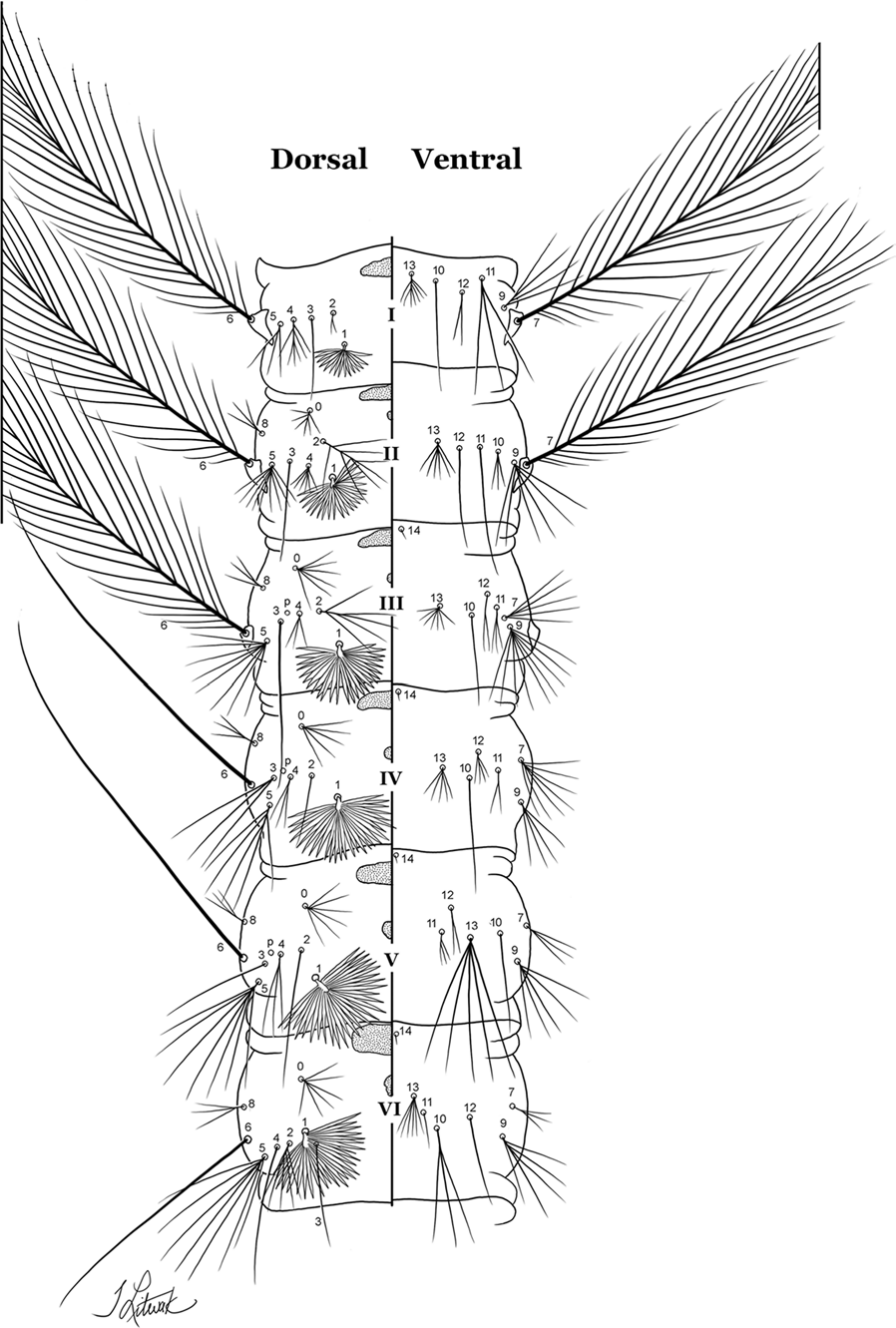
Larval abdomen of *An. goeldii*. Abdominal segments I–VI, dorsal aspect, left, ventral aspect, right

**Fig. 6:**
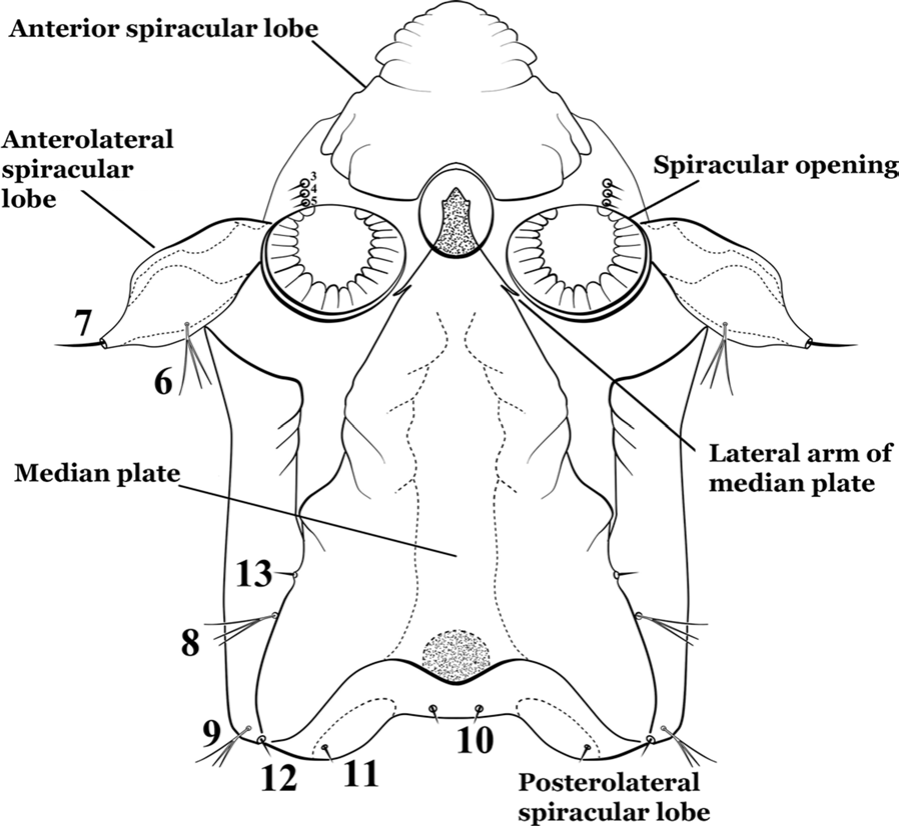
Spiracular apparatus of fourth-instar larva of *Anopheles* species, dorsal aspect

**Fig. 7:**
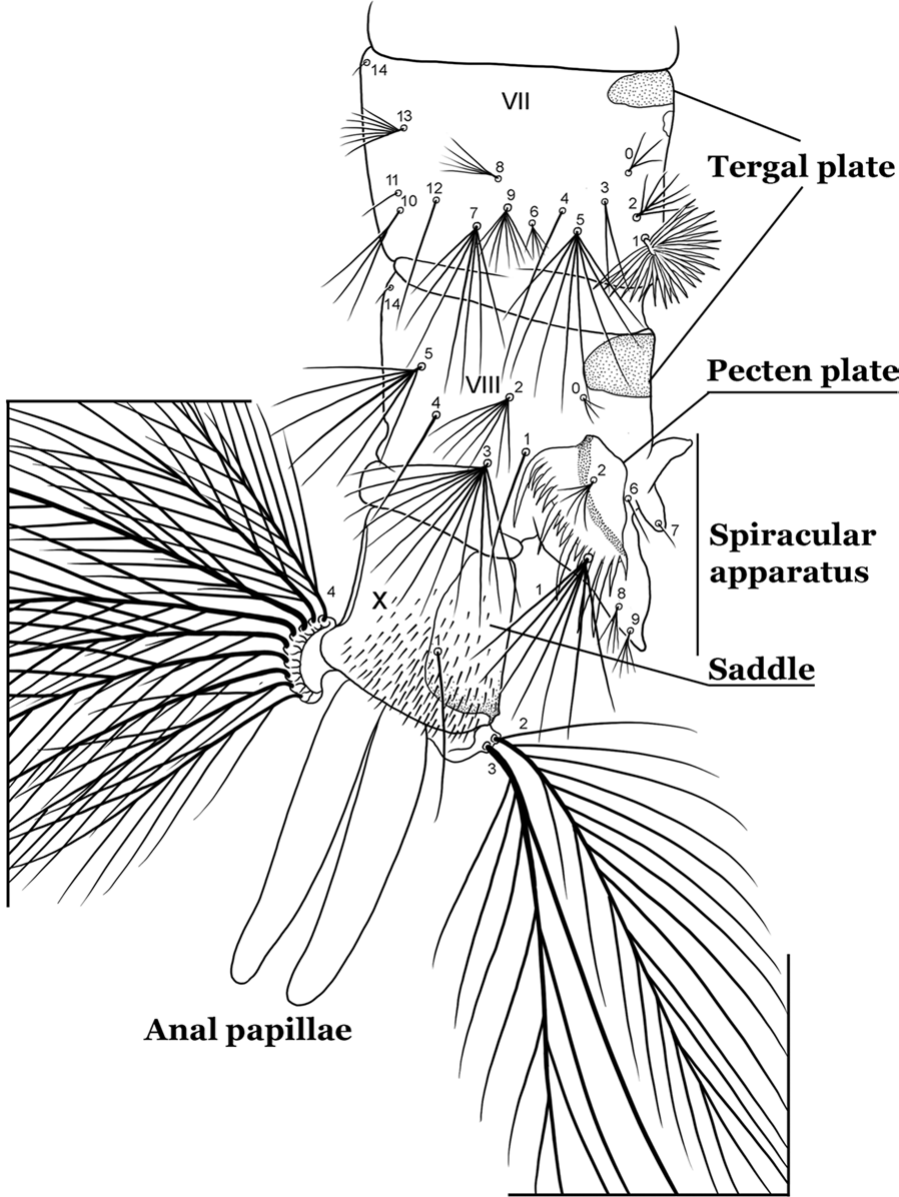
Segments VII–X of abdomen of fourth-instar larva of *An. goeldii*, lateral aspect

As in all Culicidae, the abdomen is composed of 10 segments, nine of them visible. Segments are identified by Roman numerals (I–X). The larva usually has the following pairs of setae: up to 12 on segment I, 15 on II-VII, seven on VIII, 13 on the spiracular apparatus and pecten, and four on X [5]. In the case of the spiracular apparatus and pecten, they are referenced by the letter S, to indicate that they are equivalent to the setae of the siphon in species of the subfamily Culicinae. The setal nomenclature starts with the number 0 (zero). Setae 8 and 14 are absent from segment I (Fig. 1). The following principal diagnostic setae are all dorsal or lateral. Seta 0-II–VII is the most anterior seta. It is usually poorly developed or inconspicuous, but its variability makes it useful for the identification of some species. Seta 1-I–VII is posterolateral, nearly equidistant between the midline and lateral margins (Fig. 5). In *Anopheles*, this seta, at least on segments III–VI, is usually palmate (form suggesting a palm frond or fan) or with filiform branches. In species of *Chagasia*, seta 1 is also palmate, but the leaflets are distinctive paddle-like structures with a long slender hair-like extension. Some species of *Anopheles* (found in tree holes) do not have palmate setae, or the palmate setae are not present on all segments. Palmate setae are variously developed, but the usual form is for the individual leaflets to have a short stem with many expanded leaflets. However, diagnostic variation includes having the margins smooth or strongly to moderately toothed, apices narrowed, filamentous, diamond-shaped, or truncate. Seta 6-I–VII is lateral, generally on a tubercle. These are informally called lateral setae and are often the longest setae. Seta 6-IV–VII is usually simple or with a few branches but on segments I–III as they can be plumose. Segments VIII and X bear the pecten plate and the spiracular apparatus, both of which present various structures of taxonomic value. These include the anterior spiracular lobe, a median plate, two posterolateral spiracular lobes, and two anterolateral spiracular lobes (Fig. 6). The two spiracles are found behind the anterior spiracular lobe and on the sides of the anterior margin of the median plate. In species of the subgenus *Stethomyia* Theobald, 1902, the spiracular openings are well separated and located at the base of each anterolateral lobe. Usually, the median plate has lateral wings or arms of variable development and length that are utilized in the identification of some species or groups, especially in the subgenus *Nyssorhynchus* Blanchard, 1902 (Fig. 6). The posterolateral spiracular lobes can be rounded or include posterior projections, somewhat similar to spines, as observed in *An*. *pseudopunctipennis pseudopunctipennis* Theobald, 1901. On either side of the spiracular apparatus is the pecten plate bearing pecten spines (Fig. 7), which is homologous in part with the siphon in the species of the Culicinae. It is a triangular plate with spines that resemble a comb. Frequently, variations in this structure are used for characterization of species of the subgenus *Kerteszia* Theobald, 1905; in some species the spines have an irregular arrangement, in others they have a regular arrangement of alternating long and short spines, whereas others exhibit equally-sized spines. There are 13 pairs of spiracular setae (1–13-S). Seta 1-S is generally the most developed and branched (with various simple branches). It is inserted posterior to the pecten plate below the posterolateral spiracular lobe. Seta 2-S is inserted at the base of the pecten plate. Setae 3–5-S, are generally small and indistinct, and 3-S is often only represented by an alveolus. These three setae are borne laterally on the anterior spiracular lobe. Setae 6-S and 7-S are on the proximal and distal anterolateral spiracular lobes, respectively. Setae 8-S and 9-S are on the proximal and distal lateral margins of the posterolateral spiracular lobe. Seta 10-S is borne on the internal posterior margin, while 11-S and 12-S are found on the posterior border of the lobe (Fig. 7). Seta 13-S can be of taxonomic importance, it is borne medially on the anterior margin of the internal surface of the lobe. It is generally small and somewhat stout. In the case of *An*. *darlingi* Root, 1926, it is usually much longer than the dorsal length of the saddle.

The dichotomous identification key for the genus *Anopheles*, using morphological characters of the fourth-instar larva, includes species of the subgenera *Anopheles* Meigen, 1818, *Kerteszia*, *Lophopodomyia* Antunes, 1937, *Nyssorhynchus* and *Stethomyia* of the South America. Specimens of *An. acanthotorynus*, *An*. *albertoi*, *An*. *arthuri*, *An*. *bustamantei*, *An*. *canorii*, *An*. *evandroi*, *An*. *nigritarsis*, *An*. *pseudomaculipes*, *An*. *pseudopunctipennis levicastilloi*, *An*. *pseudopunctipennis noei*, *An*. *pseudopunctipennis neghmei*, *An*. *pseudopunctipennis patersoni*, *An*. *pseudopunctipennis rivadeneirai*, *An*. *rachoui*, *An*. *sanctielii* and *An*. *striatus* cannot be identified using this key. They are poorly known, and the original descriptions were based on the morphology of the eggs, or females, or males with no further association of all life stages. In addition, *An. pseudopuntipennis* encompasses six subspecies that are morphologically similar in all life stages, including the male genitalia. Thus, specimens of these subspecies will be identified as *An*. *pseudopunctipennis*. However, it is highly recommended to use geographical localities as additional information for identification. *Anopheles striatus* belongs to the Strodei Subgroup of the Oswaldoi Group. This subgroup includes *Anopheles albertoi*, *Anopheles arthuri*, *Anopheles rondoni* (Neiva & Pinto, 1922), *Anopheles striatus* and *Anopheles strodei*. These species can be identified using DNA sequences of mitochondrial and nuclear genes [10–12], and morphological characters of the eggs, larvae, pupae, male genitalia, and females [13–15]. However, a detailed comparative morphological investigation will be necessary for description of all life stages and accurate species identification. In this key, specimens of species of the Strodei Subgroup will be identified as *An. strodei*/*An. rondoni*.

### Key for the identification of species of the genus *Anopheles* of South America based on morphological characters of the fourth-instar larvae

**Fig. 8:**
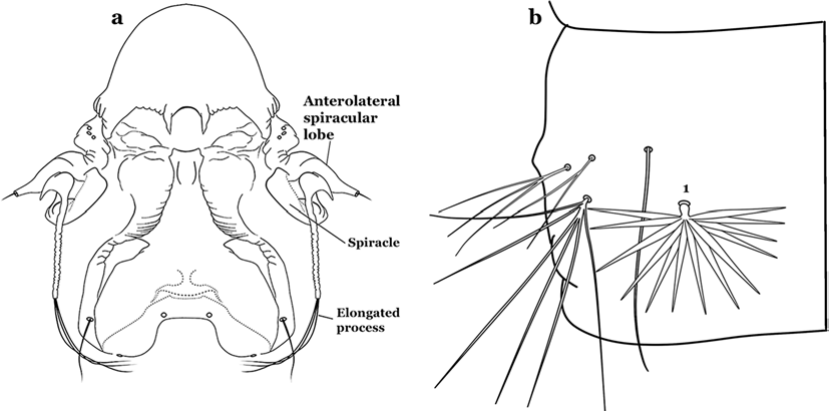
**a** *An. nimbus* (Theobald, 1902), fourth-instar larva, spiracular apparatus. **b** *An. albimanus* Wiedemann, 1820, abdominal segment, dorsal aspect, showing palmate seta 1
- Seta 1-III–VII palmate, easily visible with low magnification (Fig. 8b); anterolateral lobe of spiracular apparatus without an elongate process; spiracles not separated as above (Fig. 6)……4
- Seta 1-P single or with 2 or 3 apical branches (Fig. 9a)……*An. thomasi*

- Seta 1-III–VII small with filiform branches, not palmate, difficult to see without high magnification; anterolateral lobe of spiracular apparatus with an elongate process, ring-shaped; spiracles well separated, originating at base of anterolateral lobes (Fig. 8a)……2

**Fig. 9:**
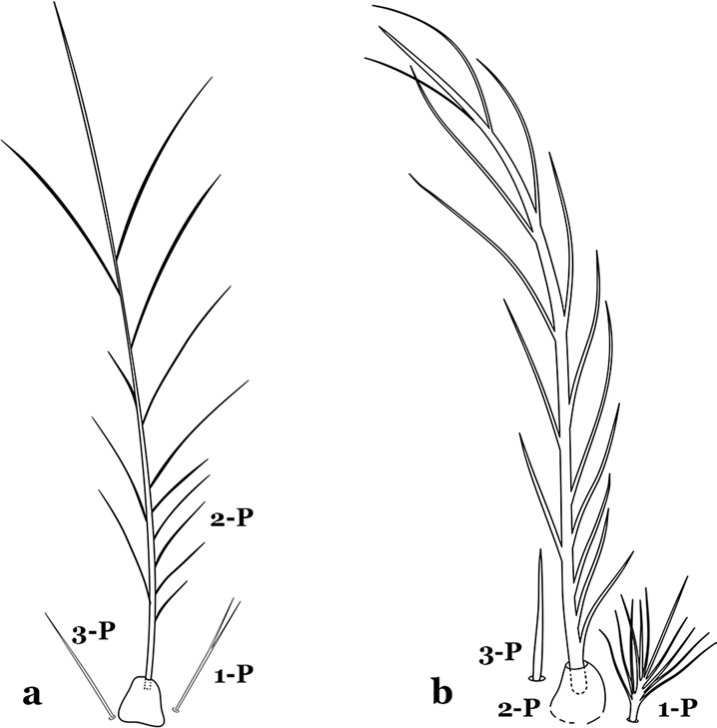
Prothoracic setae 1–3-P. **a** *An. thomasi* Shannon, 1933. **b** *An. nimbus*
- Seta 1-P with 6–14 branches (Fig. 9b)……3
- Seta 1-P with 6–8 branches (Fig. 10a)……*An*. *kompi*

**Fig. 10:**
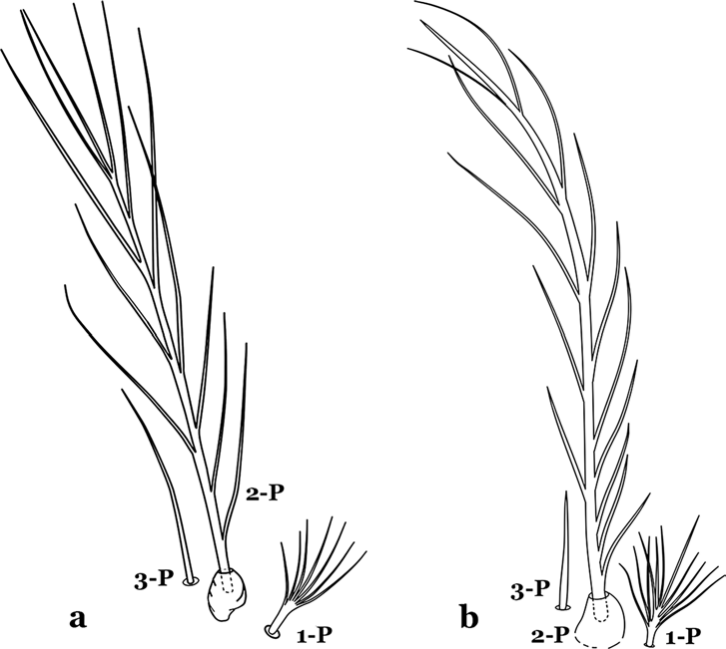
Prothoracic setae 1–3-P. **a** *An. kompi* Edwards, 1930. **b** *An. nimbus*
- Seta 1-P with 11–14 branches (Fig. 10b)……*An*. *nimbus*
- Leaflets of seta 1-II–VII smooth-sided, apices variable (Fig. 11a, b)……5

**Fig. 11:**
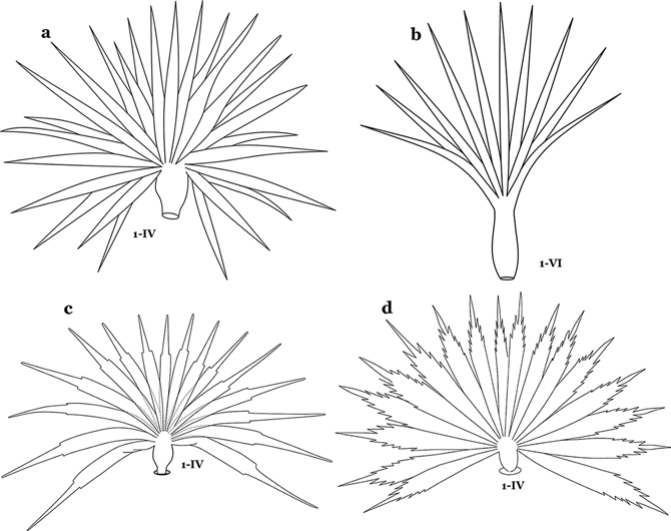
Abdominal seta 1. **a**, b *An. bellator* Dyar & Knab, 1906 seta 1-IV, VI. **c** *An. pseudopunctipennis* Theobald, 1901 seta 1-IV. **d** *An. mattogrossensis* Lutz & Neiva, 1911 seta 1-IV
- Leaflets of seta 1-II–VII with slightly serrated margins, notched or distinctly serrate on apical third (Fig. 11c, d)……42
- Setae 5–7-C single, double or branched apically, never plumose (Fig. 12a, b)……6

**Fig. 12:**
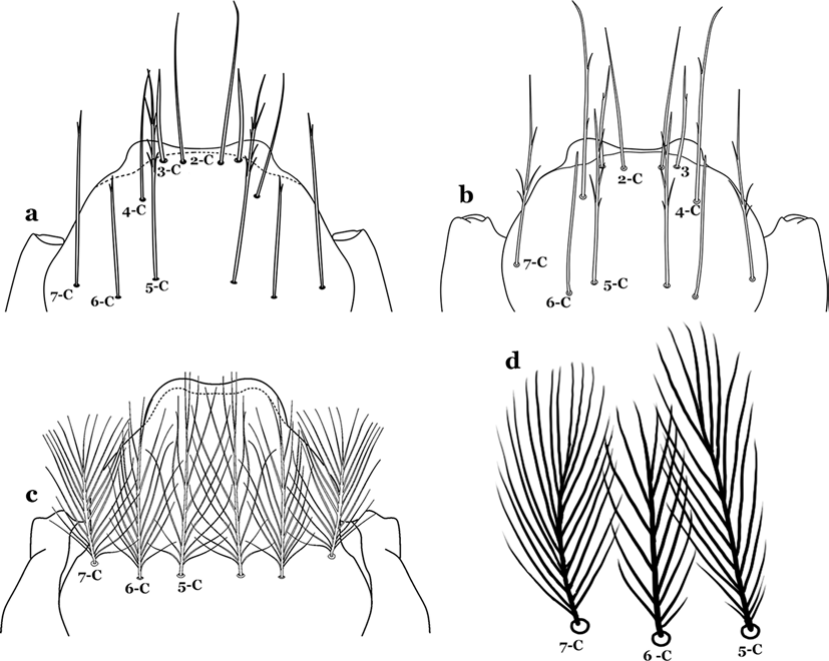
Larval head, setae 2–7-C. **a** *An. cruzii* Dyar & Knab, 1906. **b** *An. laneanus* Corrêa & Cerqueira, 1944. **c** *An. darlingi* Root, 1926, setae 5–7-C. **d** *An. goeldii*, setae 5–7-C
- Setae 5–7-C plumose (Fig. 12c, d)……17
- Seta 1-I single or branched, not palmate……7
- Seta 1-I branched, palmate (Fig. 13a, b)……13

**Fig. 13:**
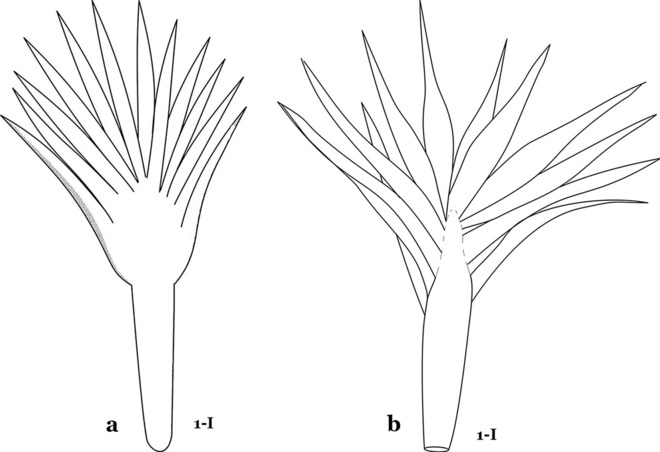
Abdominal seta 1-I. **a** *An. cruzii*. **b** *An. bellator*
- Setae 5,7-C branched apically; seta 6-C usually single (Fig. 14a)……*An*. *rollai*

**Fig. 14:**
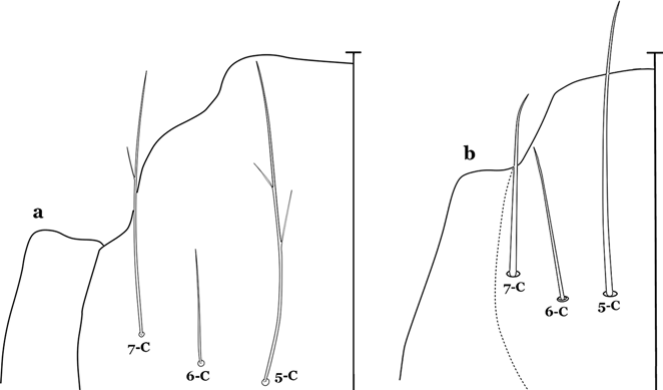
Larval head, setae 5–7-C**. a** *An. rollai* Cova Garcia, Pulido F. & Escalante de Ugueto, 1977. **b** *An. boliviensis* (Theobald, 1905)
- Setae 5,6-C usually single (Fig. 14b); seta 7-C single or double……8
- Seta 9-II–VII single (Fig. 15a); seta 11-C single (Fig. 15b)……*An*. *gonzalezrinconesi*

**Fig. 15:**
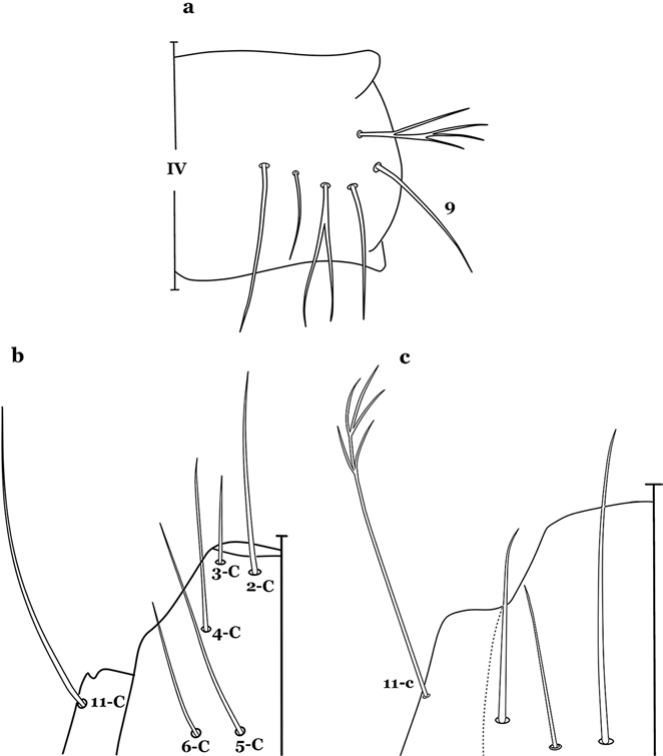
**a**, **b** *An. gonzalezrinconesi* Cova Garcia, Pulido F. & Escalante de Ugueto, 1977. **a** Abdominal seta 9-IV. **b** Head seta 11-C. **c** *An. boliviensis* Head seta 11-C
- Seta 9-II,III branched, 9-IV–VII single or branched; seta 11-C single or with 2 or 3 apical branches (Fig. 15b, c)……9
- Seta 1-II–VI with sharply pointed leaflets……*An*. *boliviensis*
- Seta 1-II–VI fan-like, with blunt-tipped leaflets spreading outward so the seta resembles a fan, or truncate (Fig. 16)……10

**Fig. 16:**
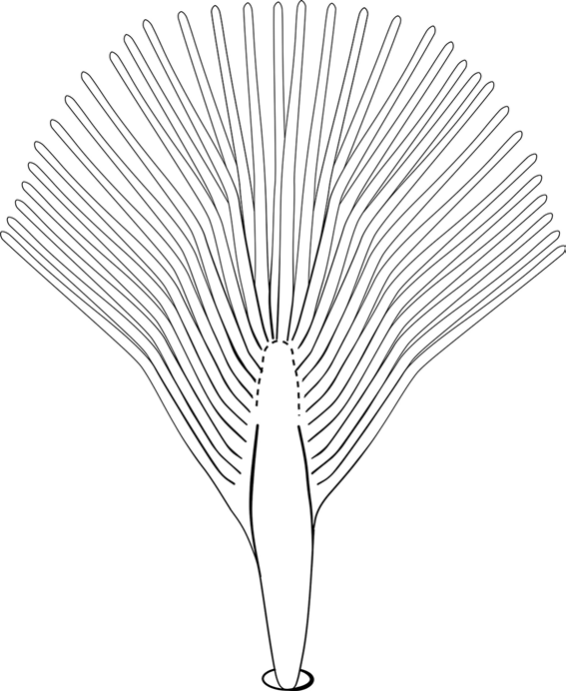
Abdominal seta 1-II–VI*. An. neivai* Howard, Dyar & Knab, 1913
- Seta 1-VII fan-like plumose (Fig. 16); seta 6-VI long, aciculate, similar to 6-III–V (Fig. 17a)……11

**Fig. 17:**
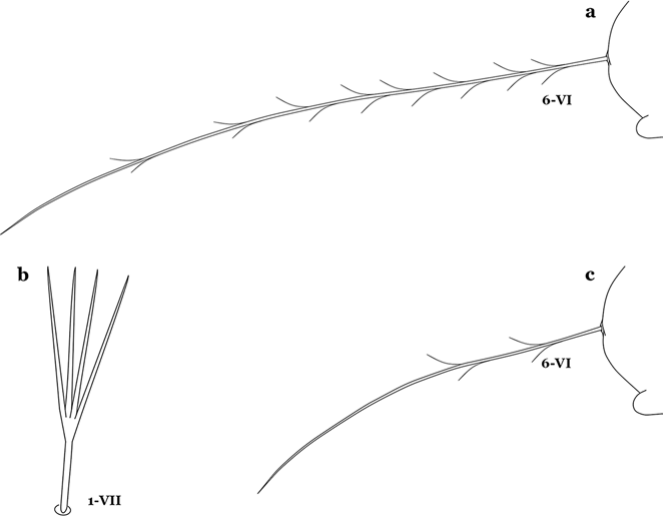
**a** *An. neivai* abdominal seta 6-VI. *An. pholidotus* Zavortink, 1973. **b** Abdominal seta 1-VII. **c** Abdominal seta 6-VI
- Seta 1-VII not palmate (Fig. 17b); seta 6-VI moderately long to long, not simple, or with a few long proximal aciculae (Fig. 17c), different from 6-III–V, with filamentous branches……12
- Seta 13-III–V usually triple and shorter than its corresponding abdominal segment (Fig. 18a)……*An. neivai*

**Fig. 18:**
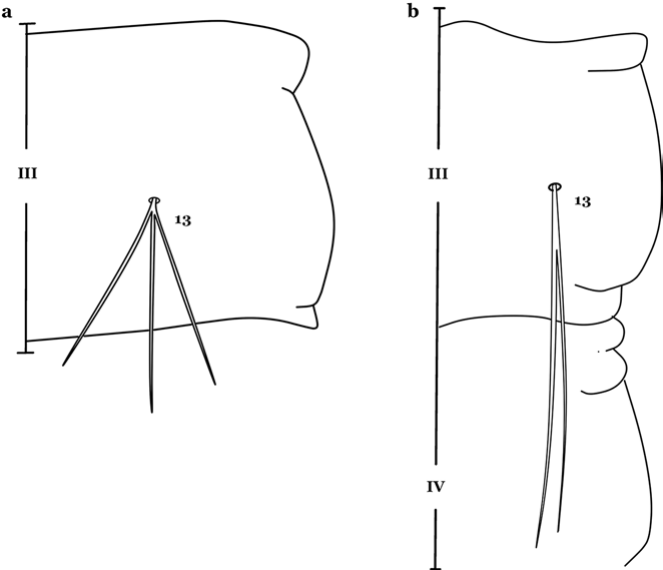
Abdominal seta 13-III. **a** *An. neivai*. **b** *An. auyantepuiensis* Harbach & Navarro, 1996
- Seta 13-III–V usually double and much longer than its corresponding abdominal segment (Fig. 18b)……*An*. *auyantepuiensis*
- Seta 3-C stout, moderately long, greater than 0.5 length of 2-C (Fig. 19a); seta 11-P well developed, longer than 0.5 length of 9-P (Fig. 19b)……*An*. *pholidotus*

**Fig. 19:**
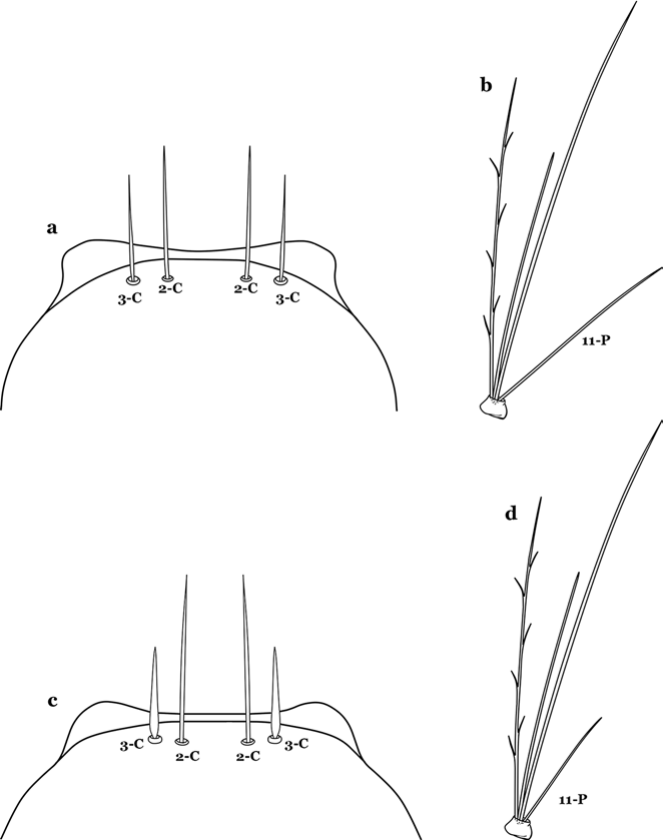
**a**, **b** *An. pholidotus.* **a** Head seta 3-C. **b** Prothoracic seta 11-P. **c**, **d** *An. lepidotus*. **c** Head seta 3-C. **d** Prothoracic seta 11-P
- Seta 3-C short and stout, spiniform, shorter than 0.5 length of 2-C (Fig. 19c); seta 11-P less developed, shorter than 0.5 length of 9-P (Fig. 19d)……*An*. *lepidotus*
- Seta 1-I with large lanceolate leaflets, similar to 1-II–VI (Fig. 20a); seta 6-VI with some long proximal aciculae (Fig. 20b)……*An*. *bambusicolus*

**Fig. 20:**
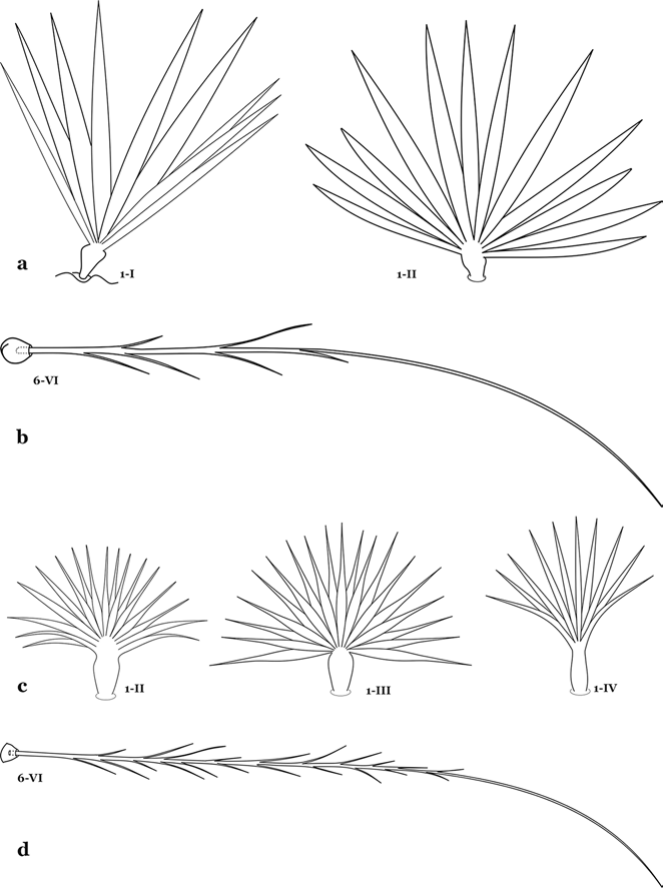
**a**, **b** *An. bambusicolus* Komp, 1937. **a** Abdominal seta 1-I, II. **b** Abdominal seta 6-IV. **c** *An. bellator*, abdominal seta 1-II–IV. **d** *An. cruzii*, abdominal seta 6-VI
- Seta 1-I–VII with narrow pointed leaflets (Fig. 20c); seta 6-VI aciculate (Fig. 20d)……14
- Seta 1-S branched (Fig. 21a)……*An*. *bellator*

**Fig. 21:**
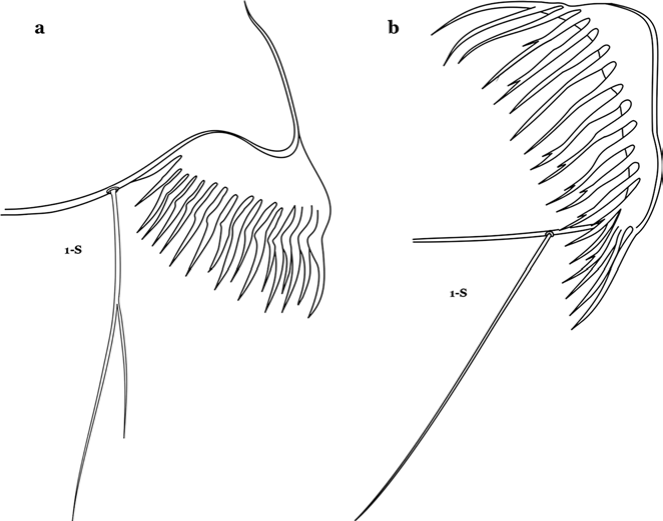
Pecten, siphonal seta 1-S. **a** *An. bellator*. **b** *An. laneanus*
- Seta 1-S single (Fig. 21b) or weakly aciculate……15
- Saddle strongly sclerotized, dark brown (Fig. 22a); living larva pale purple, more evident in fourth instars……*An*. *homunculus*

**Fig. 22:**
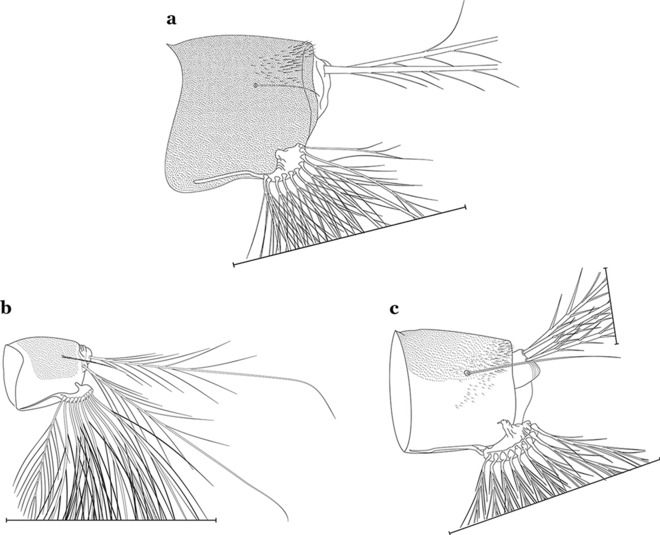
Abdominal segment X. **a** *An. homunculus* Komp, 1937. **b** *An. cruzii*. **c** *An. laneanus*
- Saddle lightly sclerotized, pale or light brown (Figs. 22b and 22c); living larva reddish, more evident in fourth instars……6
- Seta 8-C extends well past base of 6-C; seta 4-C much longer than seta 2-C; seta 2-C with or without obvious aciculae (Fig. 23a……*An*. *laneanus*

**Fig. 23:**
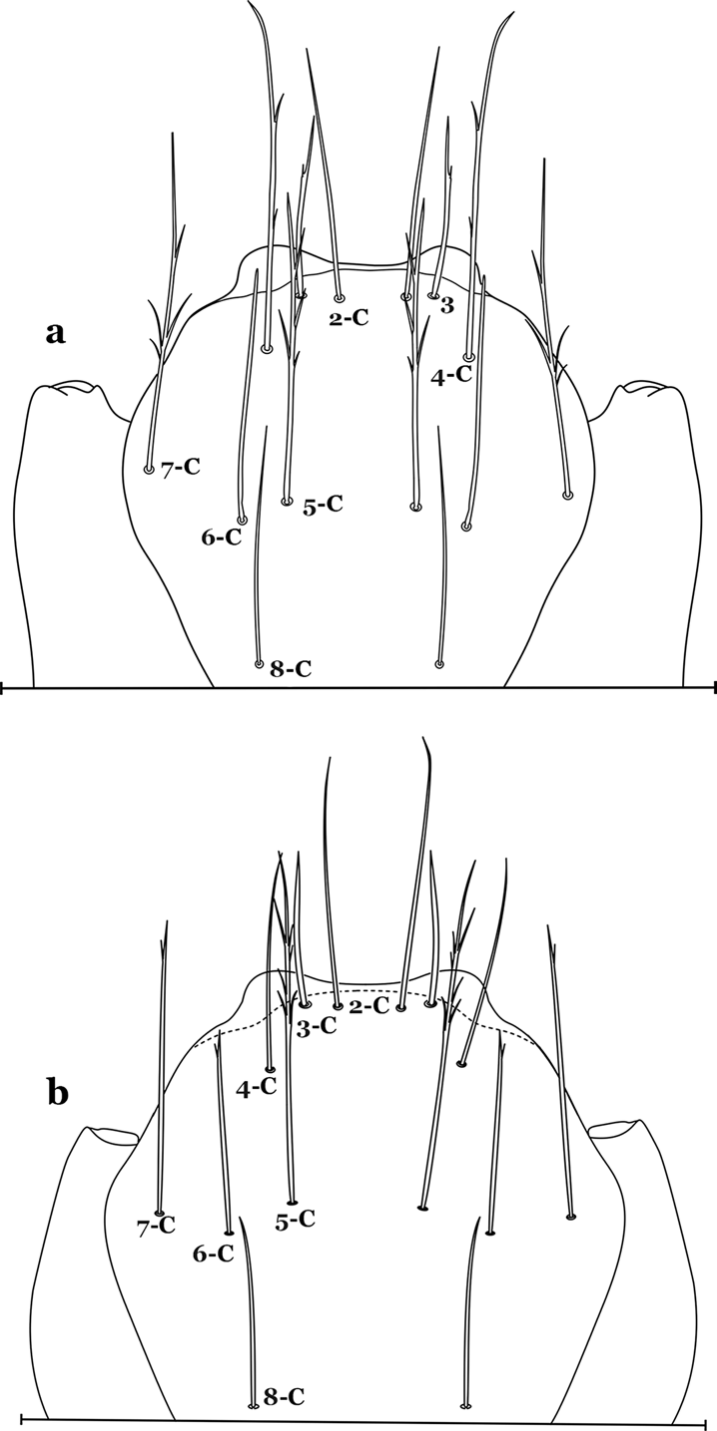
Larva head, dorsal view, setae 2–8-C. **a** *An. laneanus*. **b** *An. cruzii*
- Seta 8-C not extending well past base of 6-C; seta 4-C shorter than seta 2-C; setae 2-C or 3-C, or both, single, seta 3-C simple or with a few aciculae (Fig. 23b)…….*An*. *cruzii*
- Seta 6-IV,V single (Fig. 24a)…….18

**Fig. 24:**
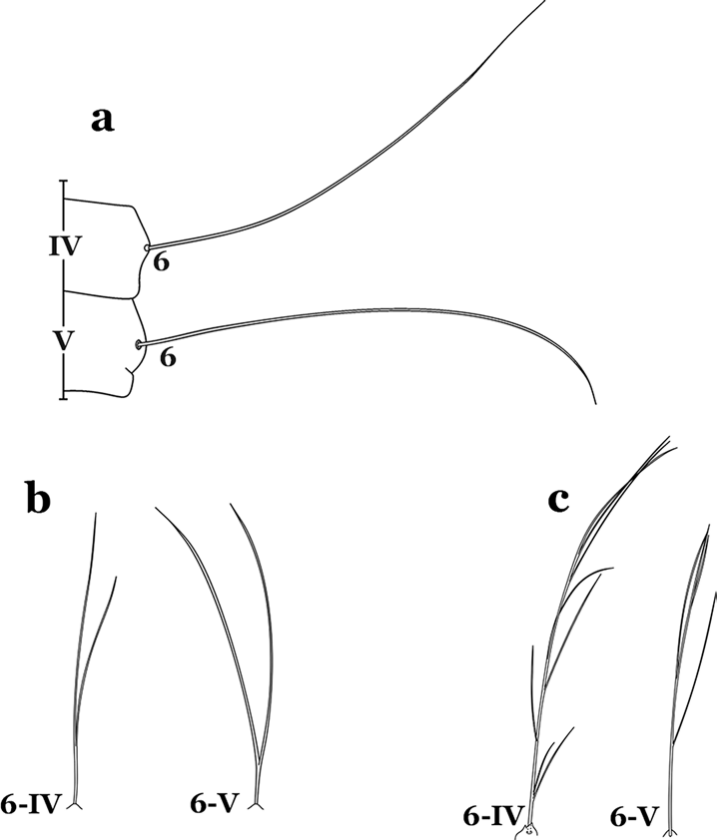
Abdominal seta 6-IV, V. **a** *An. darlingi*. **b** *An. antunesi* Galvão & Franco do Amaral, 1940. **c** *An. guarani* Shannon, 1928
- Setae 6-IV,V double or multi-branched (Fig. 24b, c)…….38
- Seta 1-P plumose, with slender branches (Fig. 25a, b)…….19

**Fig. 25:**
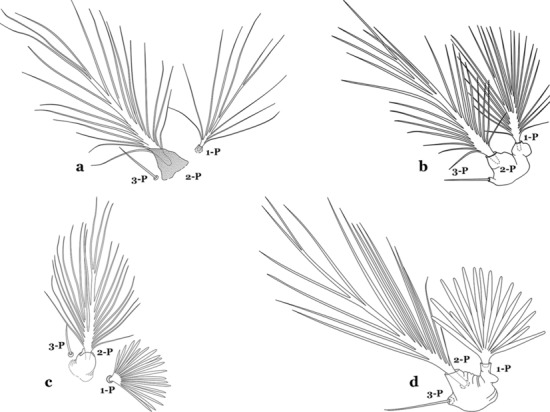
Prothoracic setae 1–3-P. **a** *An. darlingi.* **b** *An. albimanus.* **c** *An. braziliensis* (Chagas, 1907). **d** *An. marajoara* Galvão & Damasceno, 1942
- Seta 1-P fan-like, with thickened or lanceolate branches (Fig. 25c, d)…….23
- Seta 13-S well developed, long, approximately 2.2–2.5 length of saddle (Fig. 26a)…….*An*..*darlingi*

**Fig. 26:**
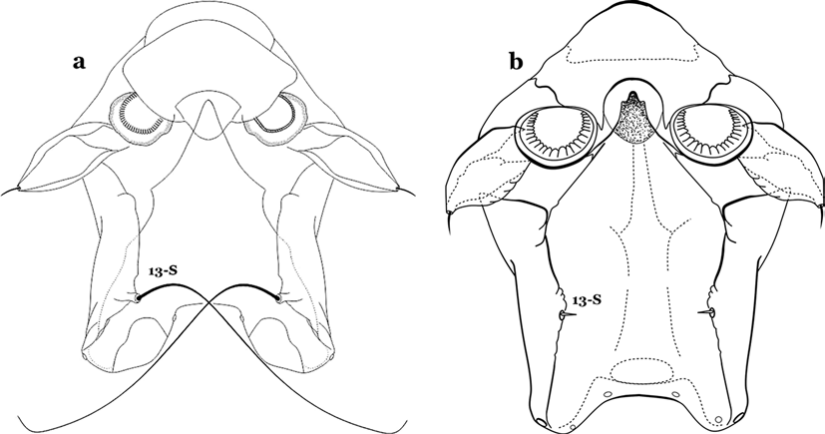
Spiracular apparatus, seta 13-S. **a** *An. darlingi*. **b** *An. albimanus*
- Seta 13-S much shorter than saddle (Fig. 26b)…….20
- Seta 3-C multi-branched distally, with long branches (Fig. 27a); clypeal index 1.35–2…….*An*. *lanei*

**Fig. 27:**
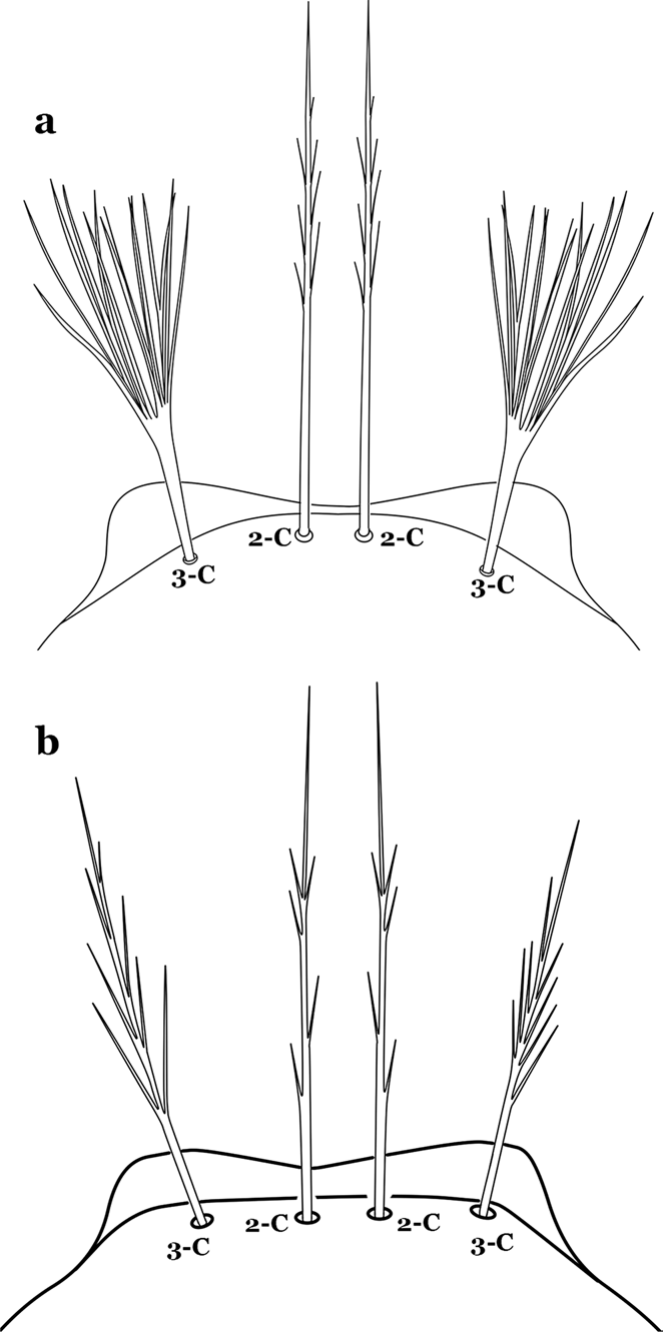
Larva head, dorsal view, seta 3-C. **a** *An. lanei* Galvão & Franco do Amaral, 1938. **b** *An. albimanus*
- Seta 3-C aciculate or with short branches (Fig. 27b); clypeal index variable…….21
- Setae 1–3-P inserted on a common tubercle (Fig. 28a); clypeal index about 1.25…….*An*. *albimanus*

**Fig. 28:**
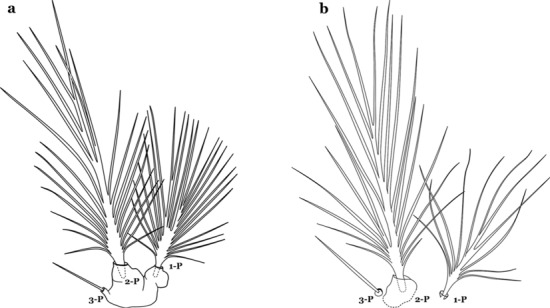
Prothoracic setae 1–3-P. **a** *An. albimanus*. **b** *An. argyritarsis* Robineau-Desvoidy, 1827
- Setae 1–3-P inserted on separate tubercles (Fig. 28b); clypeal index greater than 4.0 ….…..22
- Seta 3-T fan-like, with long narrow filamentous branches (Fig. 29a); seta 1-I pectinate with narrow, poorly sclerotized leaflets (Fig. 29b)…….*An*. *sawyeri*

**Fig. 29:**
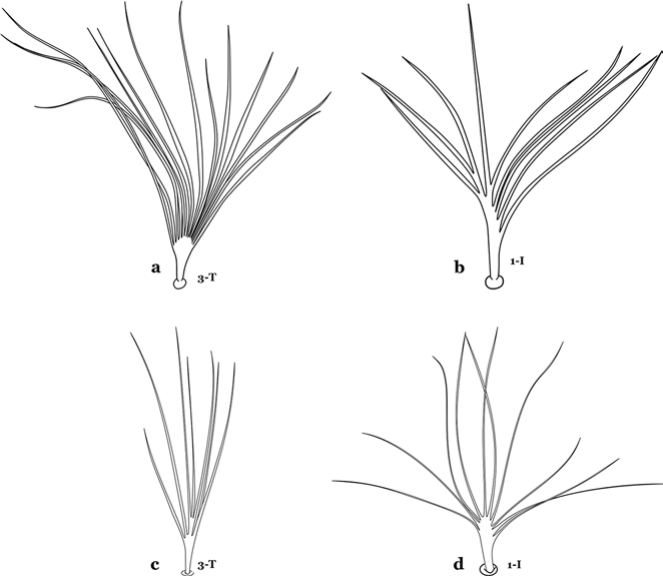
**a**, **b** *An. sawyeri* Causey, Deane, Deane & Sampaio, 1943. **a** Prothoracic seta 3-T. **b** Abdominal seta 1-I. **c**, **d** *An. argyritarsis.* **c** Prothoracic seta 3-T. **d** Abdominal seta 1-I
- Seta 3-T not fan-like, more or less pectinate, with filamentous branches (Fig. 29c); seta 1-I fan-like, with filamentous, poorly sclerotized branches (Fig. 29d)…….*An*. *argyritarsis*
- Seta 2-C moderately separated, closer together than distance between 2-C and 3-C, clypeal index usually 2.5 or more (Fig. 30a)…….24

**Fig. 30:**
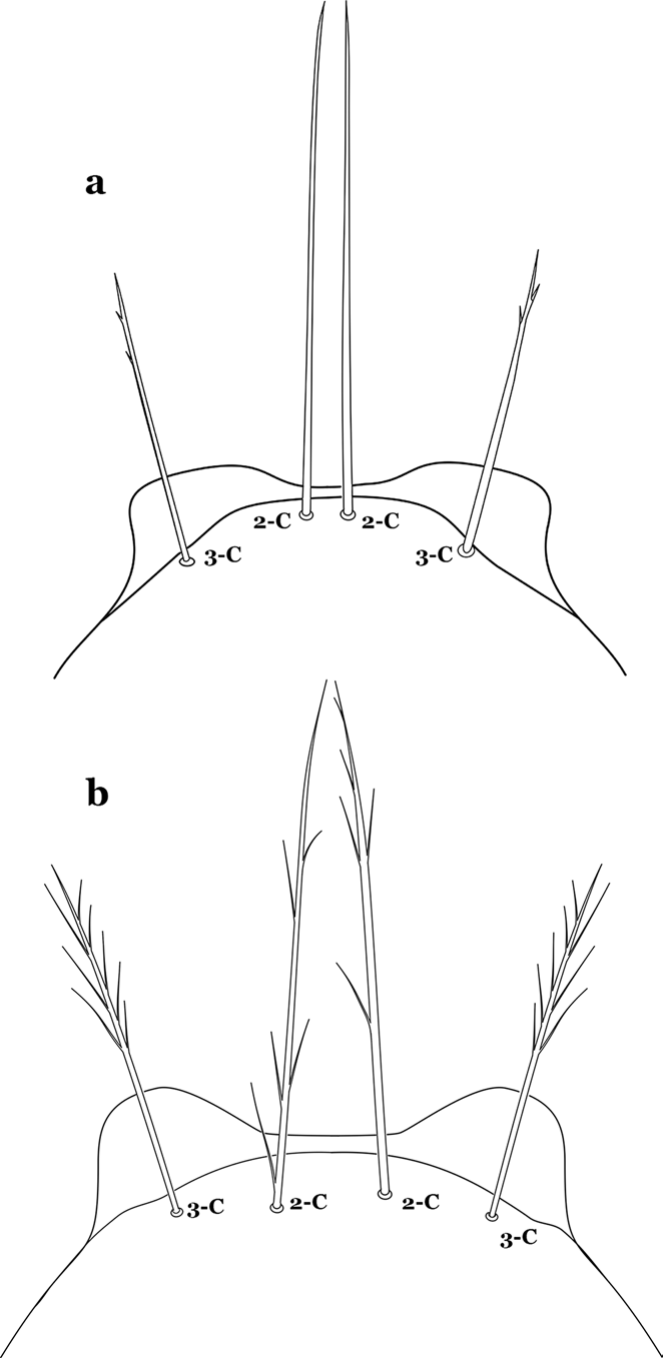
Larva head, dorsal view, setae 2, 3-C. **a** *An. braziliensis*. **b** *An. marajoara*
- Seta 2-C well separated, distance between them about equal to distance between 2-C and 3-C, clypeal index less than 2.5 (Fig. 30b)…….25
- Seta 1-P with moderately broad blunt-topped branches tips (Fig. 31a); seta 4-C long, single or double (Fig. 31b); setae 1,2-P usually inserted on a common tubercle (Fig. 31a)…….*An*. *braziliensis*

**Fig. 31:**
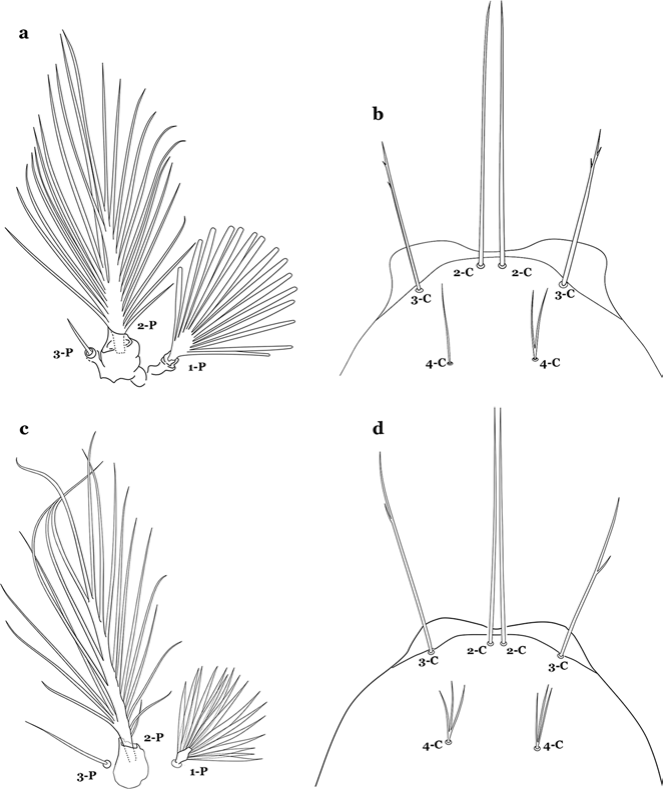
**a**, **b** *An. braziliensis*. **a** Protoracic setae 1–3-P. **b** Larva head, dorsal view, setae 2–4-C. **c**, **d** *An. strodei* Root, 1926. **c** Protoracic setae 1–3-P. **d** Larva head, dorsal view, setae 2–4-C
- Seta 1-P with narrow acuminate branches (Fig. 31c); seta 4-C short, with 1–4 branches (Fig. 31d); setae 1,2-P not inserted on a common tubercle (Fig. 31c)…….*An*. *strodei* & *An*. *rondoni*
- Seta 1–3-P usually inserted on common tubercle (Fig. 32a) or seta 1-P separate, inserted on a sclerotized tubercle of variable development……26

**Fig. 32:**
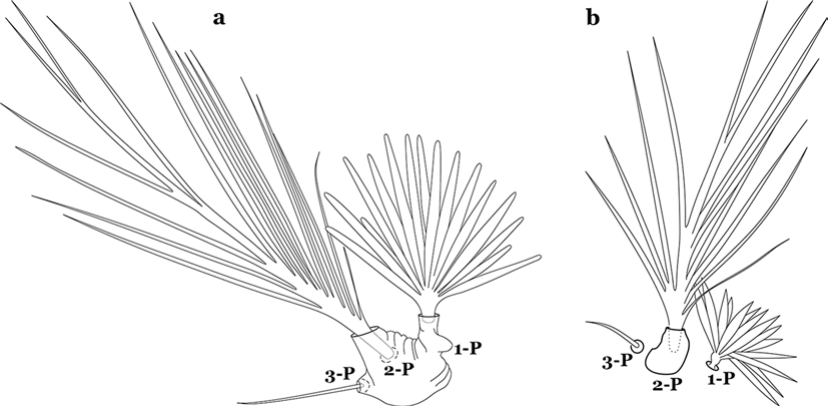
Protoracic setae 1–3-P. **a** *An. marajoara*. **b** *An. benarrochi* Gabaldon, Cova-Garcia & Lopez, 1941
- Setae 1–3-P inserted on separate tubercles (Fig. 32b)…….27
- Seta 3-C with short aciculae (Fig. 33a)…….*An*. *albitarsis*, *An*. *janconnae*, *An*. *marajoara* & *An*. *oryzalimnetes*

**Fig. 33:**
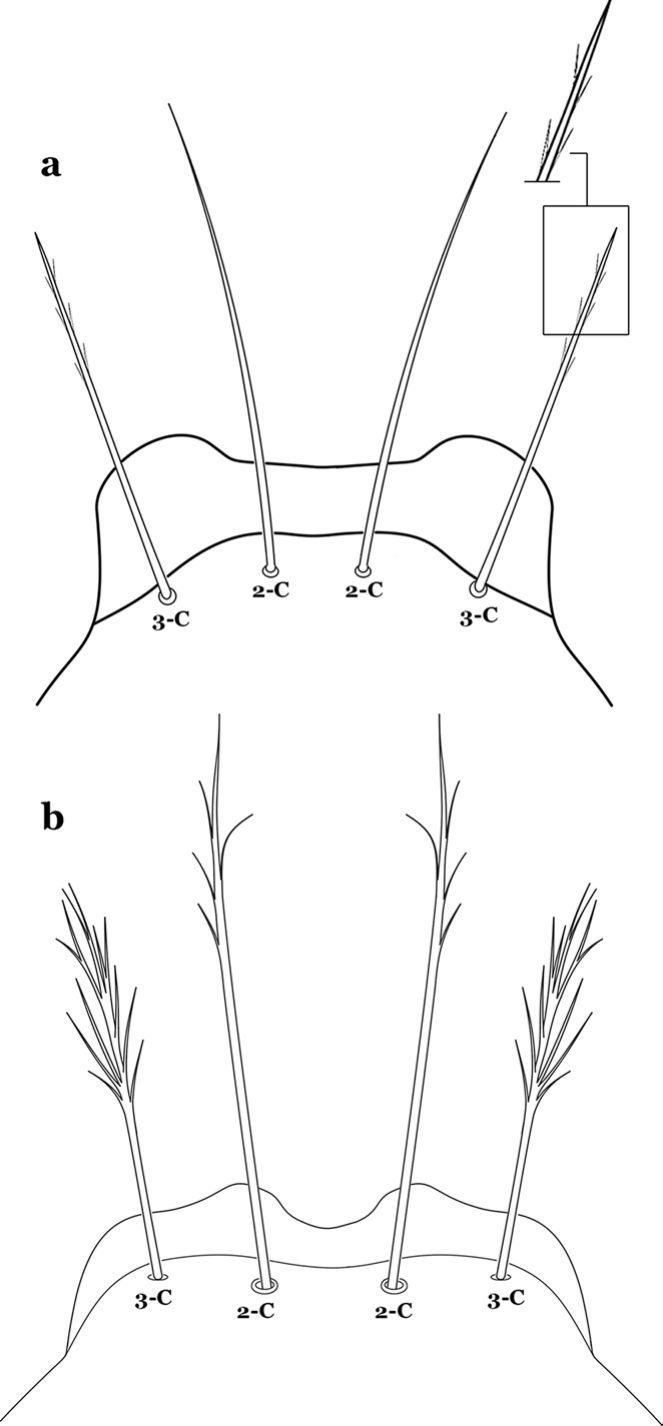
Larva head, dorsal view, setae 2, 3-C. **a** *An. albitarsis* Lynch Arribálzaga, 1878. **b** *An. deaneorum* Rosa-Freitas, 1989
- Seta 3-C branched distally (Fig. 33b)…….*An*. *deaneorum*
- Setae 2,3-C branched (Fig. 34a)…….28

**Fig. 34:**
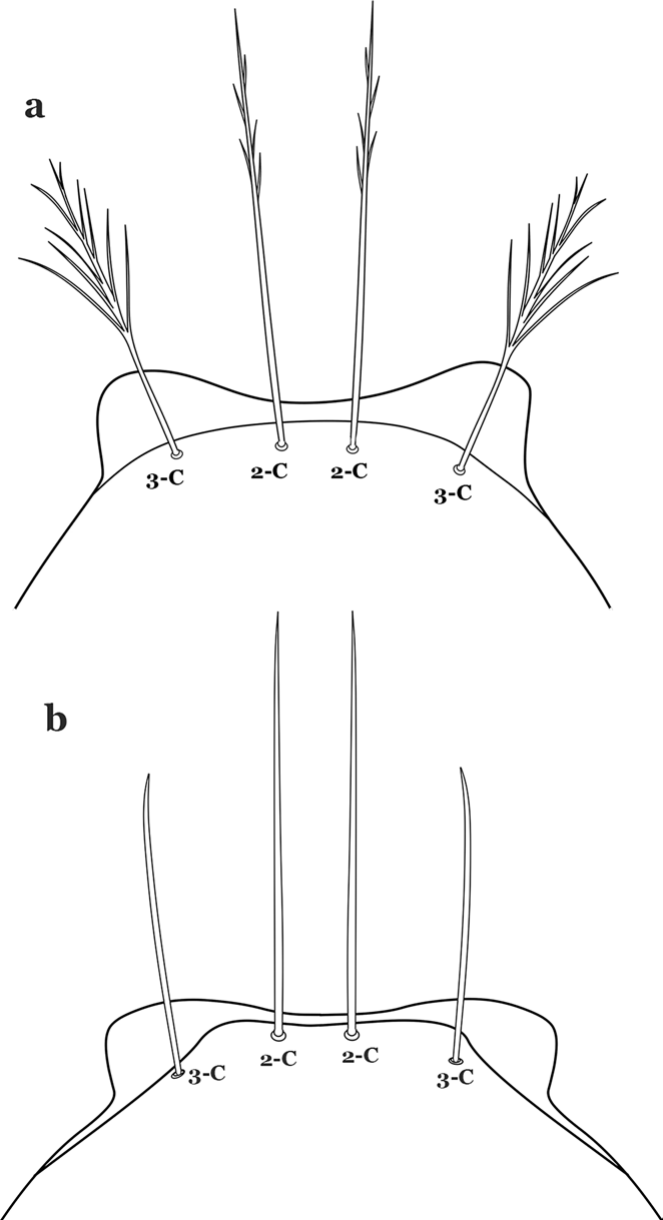
Larva head, dorsal view, setae 2, 3-C. **a** *An. benarrochi*. **b** *An. triannulatus* (Neiva & Pinto, 1922)
- Setae 2,3-C simple (Fig. 34b) or aciculate, sometimes apically branched…….30
- Seta 1-A at least 2 times longer than width of antenna at point of insertion (Fig. 35a); seta 1-X as long or slightly longer than saddle (Fig. 35b); seta 3-C branches longer than those of seta 2-C (Fig. 34a)…….*An*. *benarrochi*

**Fig. 35:**
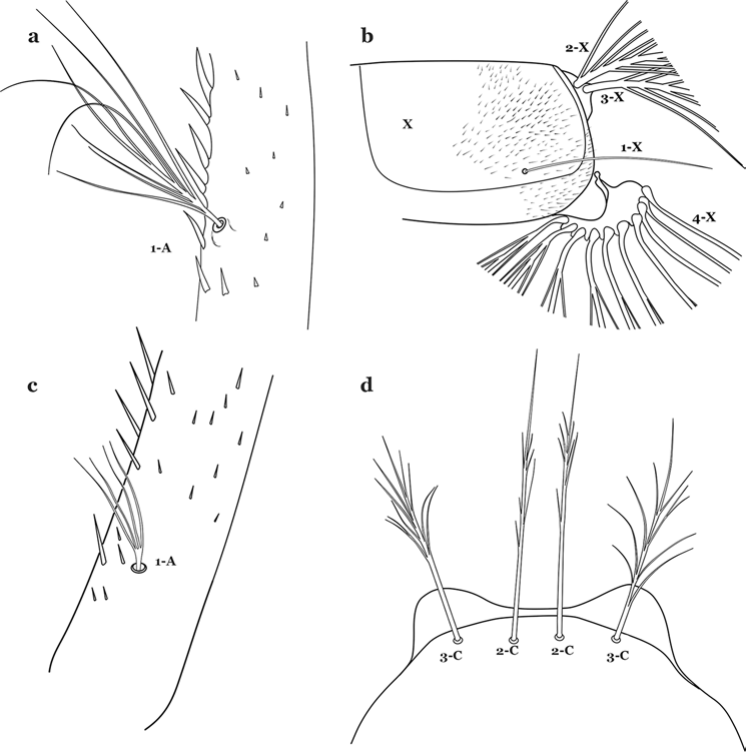
**a**, **b** *An. benarrochi*. **a** Antennal seta 1-A. **b** Abdominal seta 1-X. **c**, **d** *An. konderi* Galvão & Damasceno, 1942. **c** Antennal seta 1-A. **d** Larva head, dorsal view, setae 2, 3-C
- Seta 1-A less than twice as long as width of antenna at point of insertion (Fig. 35c), sometimes minute; seta 1-X shorter than saddle; setae 2,3-C branches similar in size or 3-C branches slightly longer than 2-C branches (Fig. 35d)…….29
- Setae 2,3-C with simple branches, rarely dendritic, branches begin on distal half (Fig. 36a); seta 1-X inserted on saddle (Fig. 36b) or in an indentation at or near ventral margin; lateral arms of median plate of spiracular apparatus minute (Fig. 36c); anal papillae usually short, approximately 0.5 length of segment X (Fig. 36b)…….*An*. *aquasalis*

**Fig. 36:**
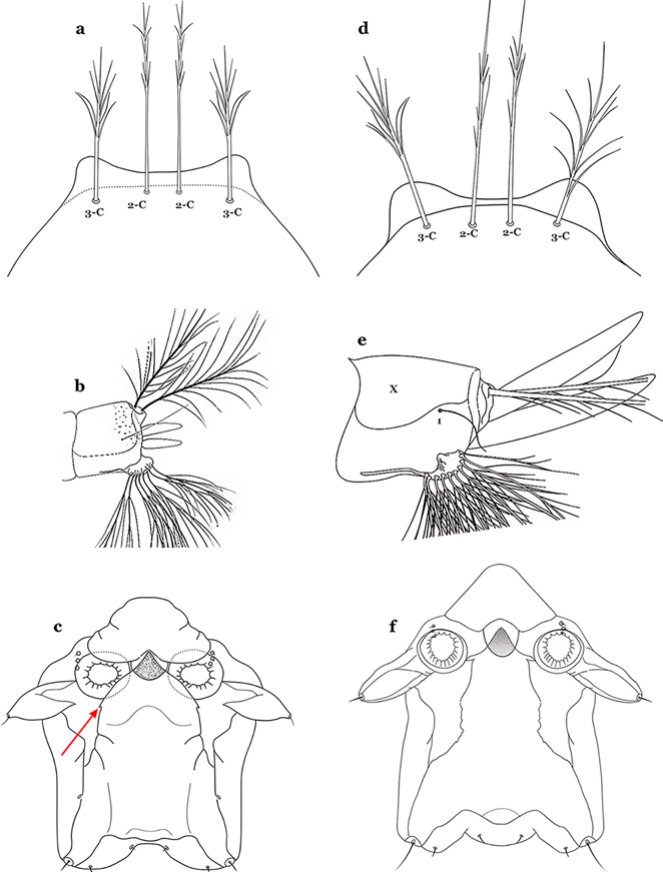
**a**–**c** *An. aquasalis* Curry, 1932. **a** Larva head, dorsal view, setae 2, 3-C. **b** Abdominal seta 1-X. **c** Spiracular apparatus. **d**-**f** *An. konderi*. **d** Larva head, dorsal view, setae 2, 3-C. **e** Abdominal seta 1-X. **f** Spiracular apparatus
- Setae 2,3-C branched, branches begin on proximal half (Fig. 36d); seta 1-X not inserted on saddle (Fig. 36e); lateral arms of median plate of spiracular apparatus moderately long (Fig. 36f); anal gills long, as long or longer than saddle (Fig. 36e)…….*An*. *oswaldoi* & *An*. *konderi*
- Lateral arm of median plate of spiracular apparatus long or moderately long, projecting laterally (Fig. 37a)…….31

**Fig. 37:**
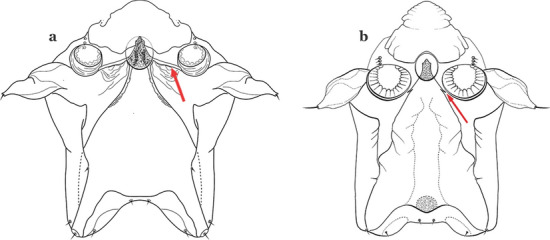
Spiracular apparatus. **a** *An. triannulatus*. **b** *An. nuneztovari* Gabaldon, 1940
- Lateral arm of median plate of spiracular apparatus absent or small, projecting caudolaterally when present (Fig. 37b)…….33
- Seta 11-I long, with 2–4 stiff branches; seta 13-I short to moderately long, usually with 4–9 branches (Fig. 38a); seta 1-P with moderately broad lanceolate branches (Fig. 38b); lateral arm of median plate of spiracular apparatus truncate at apex, stout and relatively short (Fig. 38c)…….*An*. *ininii*

**Fig. 38:**
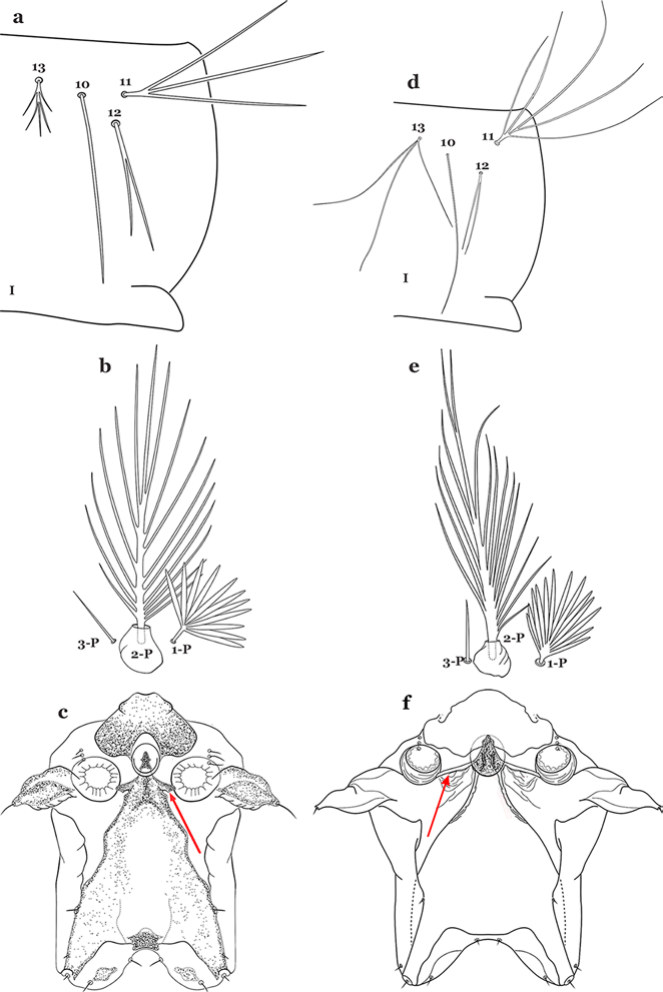
**a**–**c** *An. ininii* Senevet & Abonnenc, 1938. **a** Abdominal setae 11–13-I. **b** Prothoracic setae 1–3-P. **c** Spiracular apparatus. **d**–**f** *An. triannulatus*. **d** Abdominal setae 11–13-I. **e** Prothoracic setae 1–3-P. **f** Spiracular apparatus
- Seta 11-I long, with 3–7 branches; seta 13-I long, with 2–4 branches (Fig. 38d); seta 1-P with broad or narrow lanceolate leaflets (Fig. 38e); lateral arm of median plate of spiracular apparatus truncate at apex, slender and moderately long to long (Fig. 38f)…….32
- Seta 1-P with narrow lanceolate branches (Fig. 39a); lateral arm of median plate of spiracular apparatus long and slender (Fig. 39b)…….*An*. *triannulatus*

**Fig. 39:**
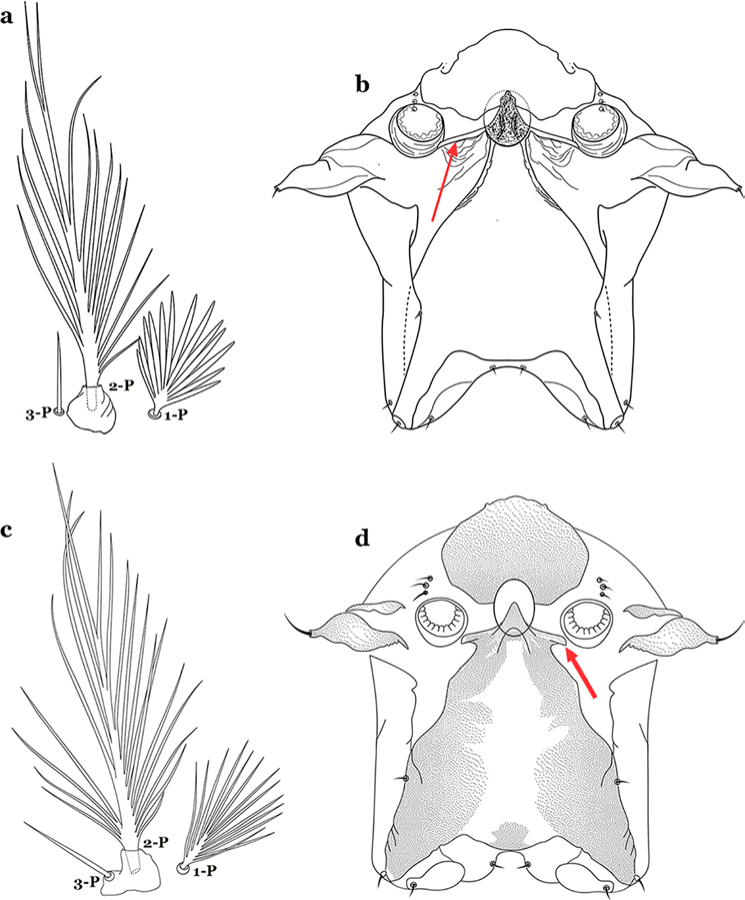
**a**, **b** *An. triannulatus*. **a** Prothoracic setae 1–3-P. **b** Spiracular apparatus. **c**, **d** *An. halophylus* Silva do Nascimento & Lourenço-de-Oliveira, 2002. **c** Prothoracic setae 1–3-P. **d** Spiracular apparatus
- Seta 1-P with narrow acuminate branches (Fig. 39c); lateral arm of median plate of spiracular apparatus shorter and stouter (Fig. 39d)…….*An*. *halophylus*
- Seta 11-I moderately long, with 3–7 branches; seta 13-I long, with 2–4 branches (Fig. 40a); seta 1-P with narrow acuminate branches (Fig. 40b)…….*An*. *halophylus*

**Fig. 40:**
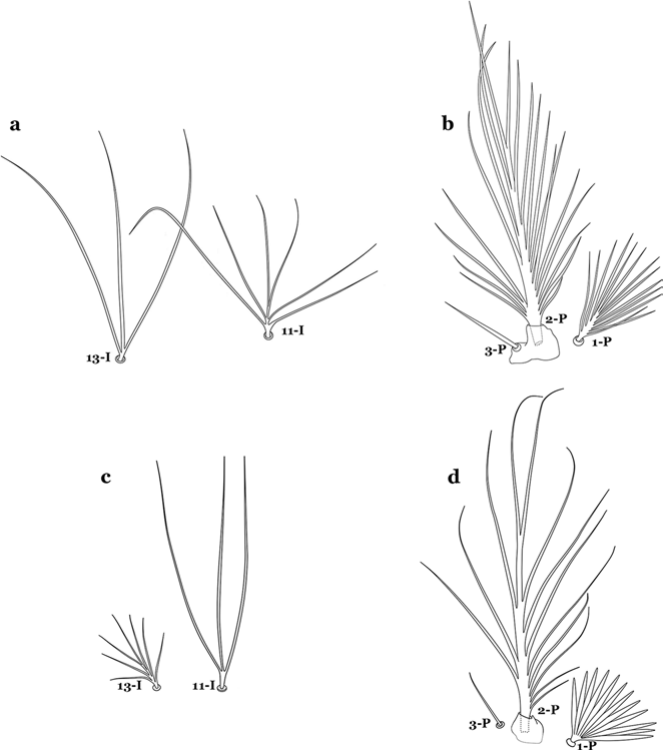
**a**, **b** *An. halophylus*. **a** Abdominal setae 11,13-I. **b** Prothoracic setae 1–3-P. **c**, **d** *An. nuneztovari*. **c** Abdominal setae 11,13-I. **d** Prothoracic setae 1–3-P
- Seta 11-I very long, with 2–4 branches; seta 13-I small, usually with more than 3 branches (Fig. 40c); seta 1-P with narrow to broad lanceolate branches (Fig. 40d)…….34
- Seta 4-C single or double, moderately long, usually extending beyond base of 2-C (Fig. 41a)…….35

**Fig. 41:**
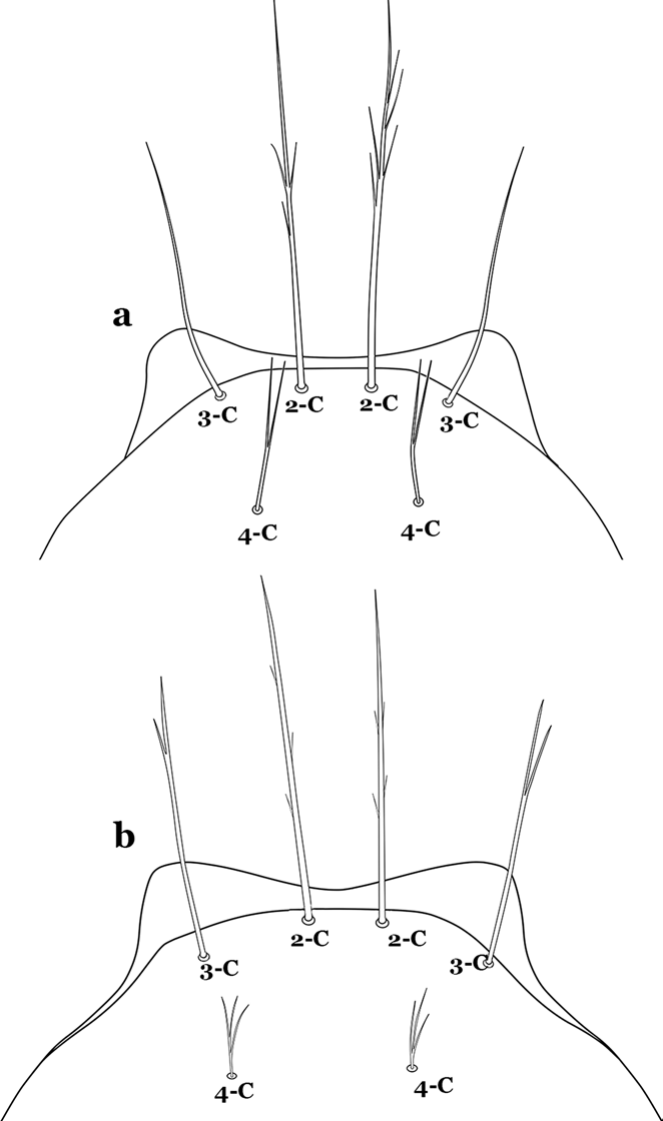
Larva head, dorsal view, setae 2–4-C. **a** *An. nuneztovari*. **b** *An. rangeli* Gabaldon, Cova-Garcia & Lopez, 1940
- Seta 4-C short, branched from base, usually not extending as far as base of 2-C (Fig. 41b)…….36
- Seta 0-II short, approximately 0.5 or less length of leaflets of seta 1-II, 1–3-branched (Fig. 42a); seta 4-C 0.7 length of 3-C (Fig. 42b)…….*An*. *dunhami* & *An*. *trinkae*

**Fig. 42:**
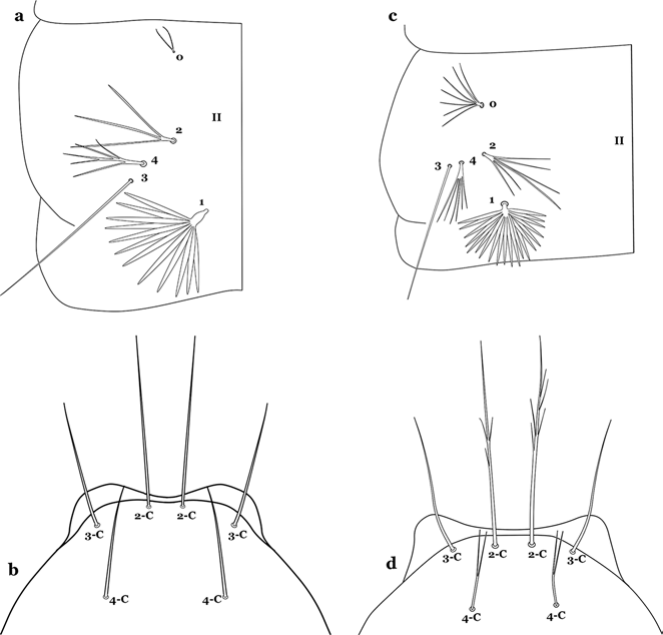
**a**, **b** *An. trinkae* Faran, 1979. **a** Abdominal setae 0–4-II. **b** Larva head, dorsal view, setae 2–4-C. **c**, **d** *An. nuneztovari*. **c** Abdominal setae 0–4-II. **d** Larva head, dorsal view, setae 2–4-C
- Seta 0-II subequal or longer than length of leaflets of 1-II, 5–8-branched (Fig. 42c); seta 4-C 0.3–0.6 length of 3-C (Fig. 42d)…….*An*. *goeldii* & *An*. *nuneztovari*
- Setae 2,3-C aciculate, with short, fine branches (Fig. 43a)…….*An*. *rangeli*

**Fig. 43:**
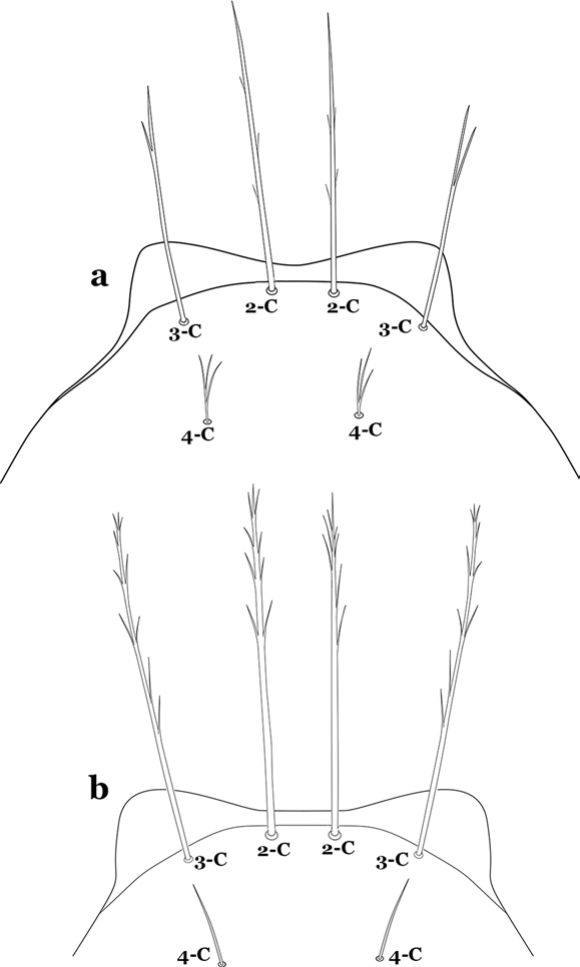
Larva head, dorsal view, setae 2–4-C. **a** *An. rangeli*. **b** *An. evansae* (Brèthes, 1926)
- Setae 2,3-C with short thick aciculae (Fig. 43b), or long fine branches…….37
- Seta 1-P with short, moderately broad branches, never narrow (Fig. 44a); lateral arm of median plate of spiracular apparatus small, or more strongly developed and discernable from median plate (Fig. 44b); seta 1-X inserted on (Fig. 44c) or outside saddle…….*An*. *galvaoi*

**Fig. 44:**
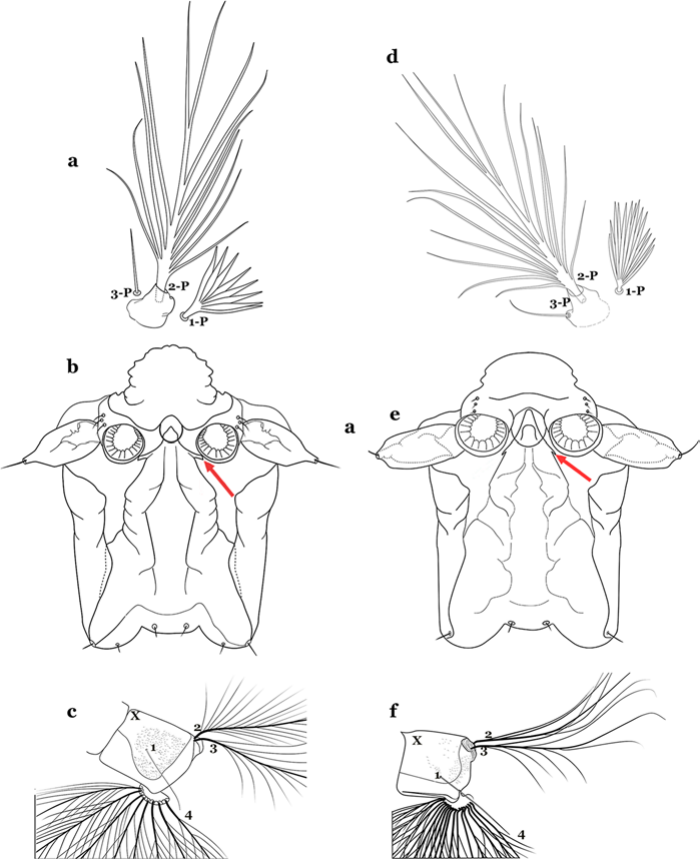
**a**–**c** *An. galvaoi* Causey, Deane & Deane, 1943. **a** Prothoracic setae 1-3-P. **b** Lateral arm of median plate of spiracular apparatus. **c** Abdominal segment X. **d**–**f** *An. evansae*. **d** Prothoracic setae 1-3-P. **e** Spiracular apparatus. **f** Abdominal segment X
- Seta 1-P with long narrow branches (Fig. 44d); lateral arm of median plate of spiracular apparatus minute (Fig. 44e); seta 1-X inserted on saddle (Fig. 44f)…….*An*. *evansae*
- Seta 6-IV–VI double (Fig. 45a)…….39

**Fig. 45:**
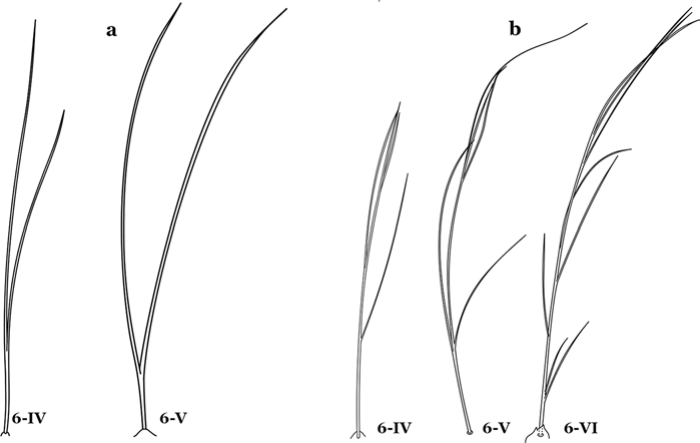
Abdominal seta 6-IV-VI. **a** *An. antunesi.* **b** *An. guarani* Shannon, 1928
- Seta 6-IV–VI multi-branched (Fig. 45b)…….40
- Seta 14-P long, extending beyond collar when specimen is mounted on a microscope slide (Fig. 46a); seta 4-C strongly developed, aciculate, extending to base of 2-C (Fig. 46b); seta 8-C branched (Fig. 46c)…….*An*. *antunesi*

**Fig. 46:**
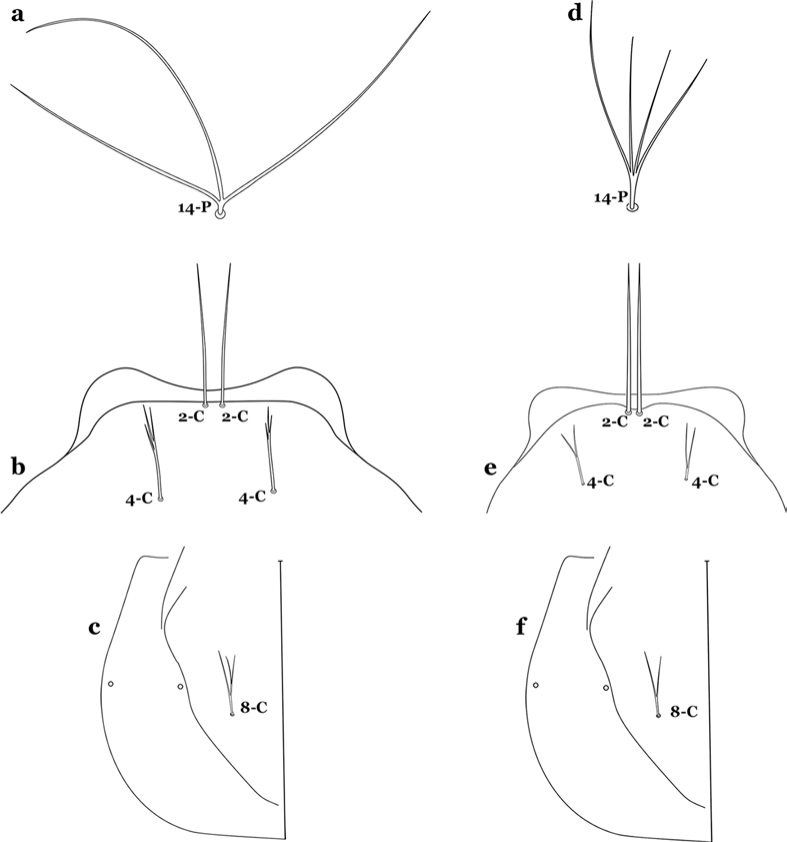
**a**–**c** *An. antunesi*. **a** Prothoracic seta 14-P. **b** Larva head, dorsal view, setae 2,4-C. **c** Seta 8-C. **d**–**f** *An. lutzii* Cruz, 1901. **d** Prothoracic seta 14-P. **e** Larva head, dorsal view, setae 2,4-C. **f** Seta 8-C
- Seta 14-P moderately short, never reaching posterior end of head (Fig. 46d); seta 4-C double, not extending as far as base of 2-C (Fig. 46e); seta 8-C double (Fig. 46f)…….*An*. *lutzii*
- Spiracular openings narrow, median plate with a heavily sclerotized mesal area, dark brown, paler laterally to posterior edge of plate (Fig. 47a)…….*An*. *guarani*

**Fig. 47:**
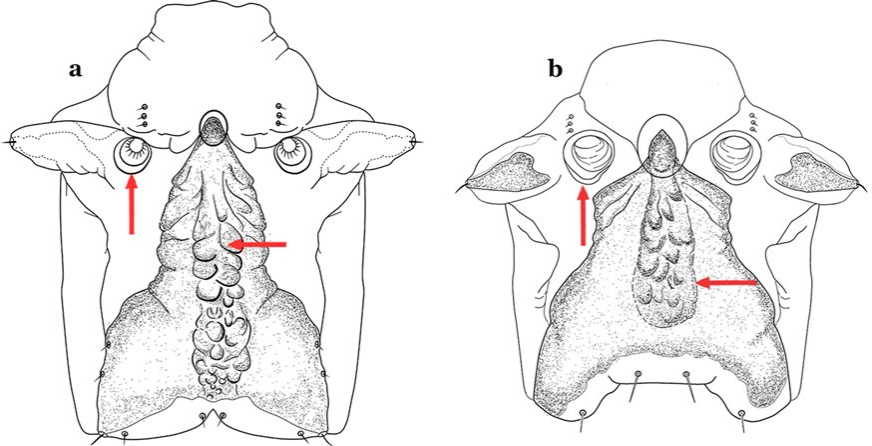
Spiracular apparatus. **a** *An. guarani*. **b** *An. parvus* (Chagas, 1907)
- Spiracular openings large, median plate with a heavily sclerotized mesal area, or not uniformly sclerotized to posterior edge of plate (Fig. 47b)…….41
- Median plate of spiracular apparatus with a heavily sclerotized mesal area (Fig. 48a); seta 1-II–VII well developed, with a short broad main stem (Fig. 48b); seta 1-A length less than twice width of antenna at point of insertion (Fig. 48c)…….*An*. *parvus*

**Fig. 48:**
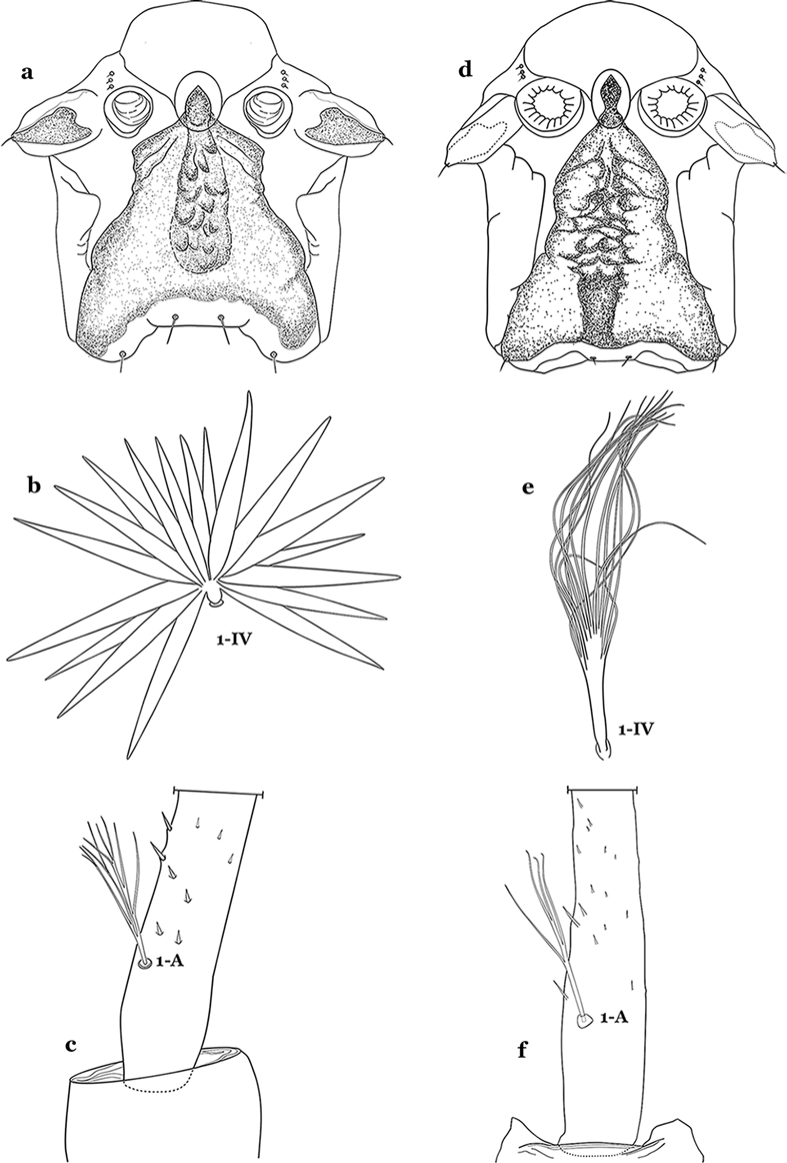
**a**–**c** *An. parvus*. **a** Spiracular apparatus. **b** Abdominal seta 1-IV. **c** Antennal seta 1-A. **d**–**f** *An. pristinus* Nagaki & Sallum, 2010. **d** Spiracular apparatus. **e** Abdominal seta 1-IV. **f** Antennal seta 1-A
- Median plate of spiracular apparatus with a strongly sclerotized mesal area posteriorly (Fig. 48d); seta 1-II–VII hyaline with a narrow long main stem (Fig. 48e); seta 1-A length more than twice width of antenna at point of insertion (Fig. 48f)…….*An*. *pristinus*
- Posterolateral lobe of spiracular apparatus with a long caudal spine (Fig. 49a)…….*An*. *pseudopunctipennis*

**Fig. 49:**
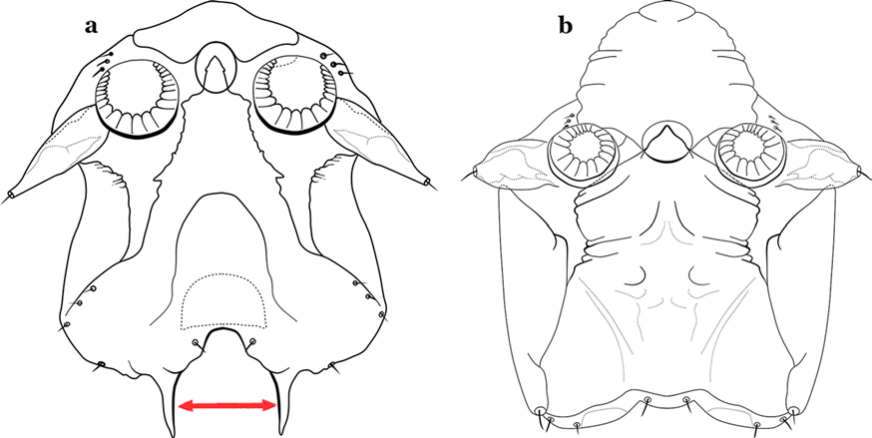
Spiracular apparatus. **a** *An. pseudopunctipennis*. **b** *An. pictipennis* (Philippi, 1865)
- Posterolateral lobe of spiracular apparatus without a long caudal spine (Fig. 49b)…….43
- Leaflets of seta 1-II–VII indistinctly serrate (Fig. 50a)…….44

**Fig. 50:**
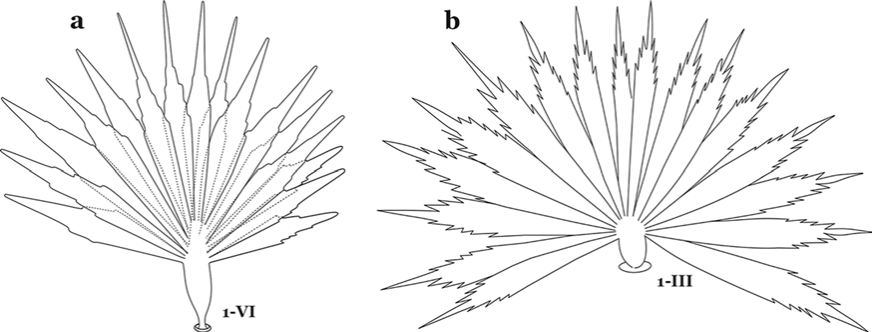
Larval abdominal seta 1. **a** *An. atacamensis* González & Sallum, 2010. **b** *An. mattogrossensis*
- Leaflets of seta 1-II–VII distinctly notched or serrate (Fig. 50b)…….46
- Seta 4-C long, reaching beyond anterior margin of head, nearly as long or as long as 2-C or 3-C; setae 2-C well separated, as close to each other as each is to 3-C; clypeal index about 1.0 (Fig. 51a)…….45

**Fig. 51:**
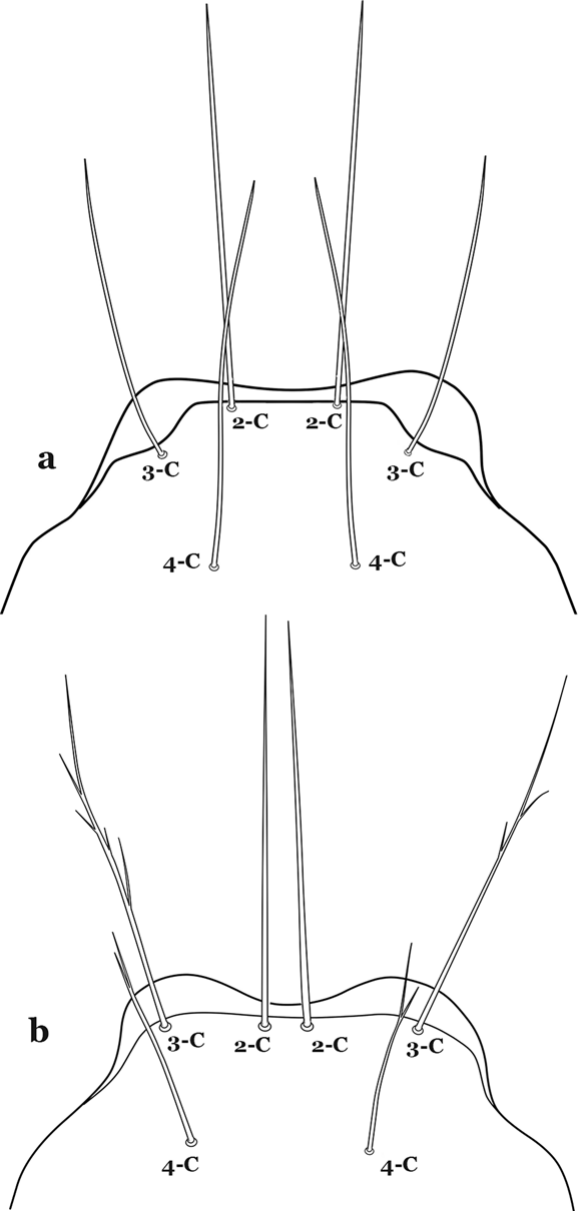
Larva head, dorsal view, setae 2–4-C. **a** *An. atacamensis*. **b** *An. oiketorakras* Osorno-Mesa, 1947
- Seta 4-C short to moderately long, not reaching beyond anterior margin of head, or not subequal to 2-C or 3-C; setae 2-C very close together, closer to another than either each is to 3-C; clypeal index greater than 1.5 (Fig. 51b)…….*An*. *gomezdelatorrei* & *An*. *oiketorakras*
- Insertion of seta 7-C in line (transverse sense) with point of insertion of setae 5-C and 6-C; seta 5-C extends to or beyond bases of 2-C or 3-C (Fig. 52a); seta 3-P inserted on same tubercle as 2-P (Fig. 52b)…….*An*. *atacamensis*

**Fig. 52:**
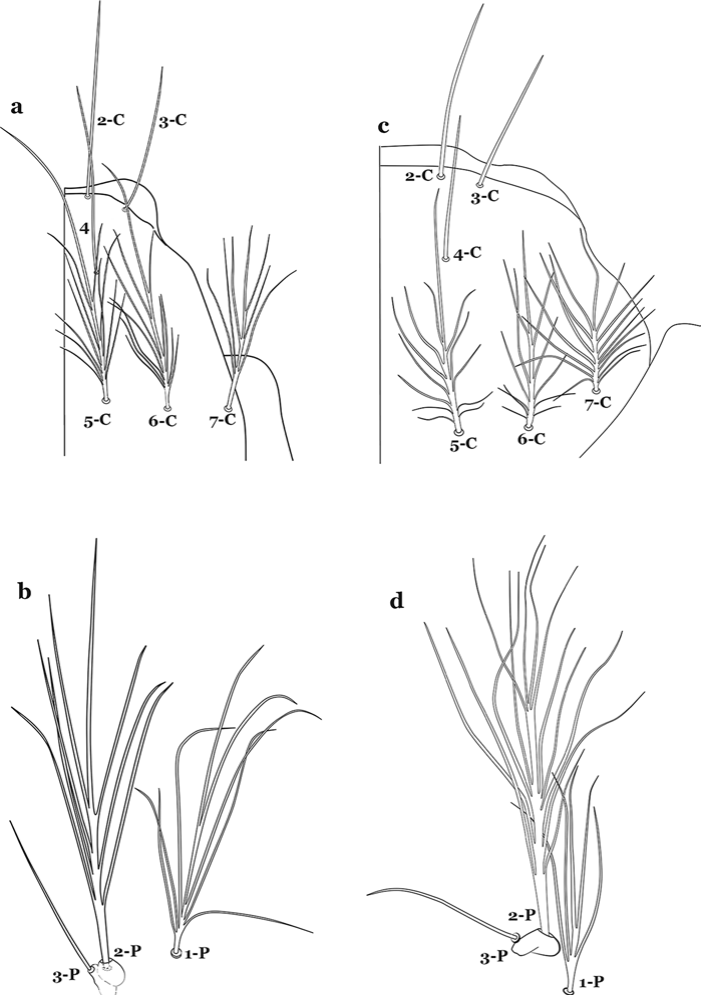
**a**, **b** *An. atacamensis*. **a** Larva head, dorsal view, setae 2–7-C. **b** Prothoracic setae 1–3-P. **c**, **d** *An. pictipennis.* **c** Larva head, dorsal view, setae 2–7-C. **d** Prothoracic setae 1–3-P
- Insertion of seta 7-C anterior of line (transverse sense) of points of insertion of setae 5-C and 6-C; seta 5-C extends to or nearly to bases of 2-C or 3-C (Fig. 52c); seta 3-P not inserted on a tubercle with 2-P (Fig. 52d)…….*An*. *pictipennis*
- Setae 2,3-A sharply pointed (Fig. 53a)…….47

**Fig. 53:**
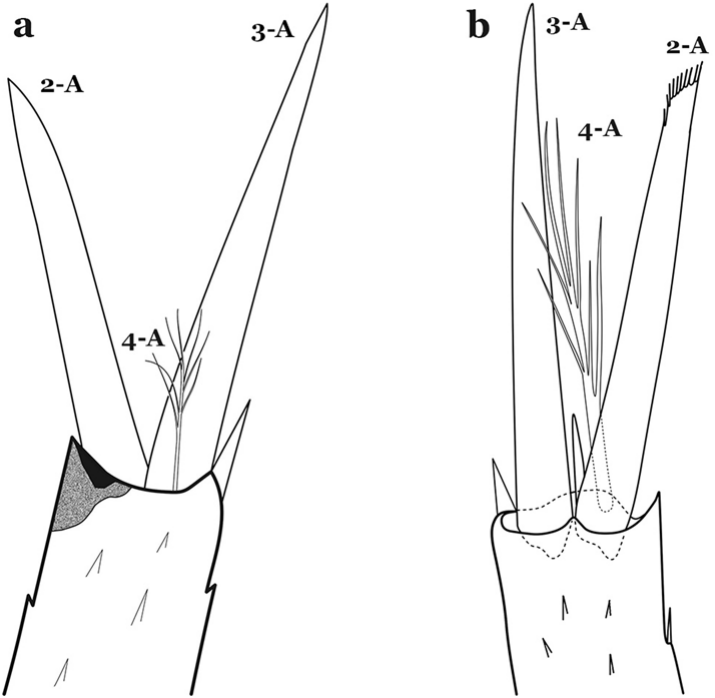
Antennal setae 2–4-A. **a** *An. atacamensis*. **b** *An. calderoni* Wilkerson, 1991
- Setae 2-A or 3-A truncate, other sharply pointed (Fig. 53b)…….55
- Seta 9-P single or double (Fig. 54a)…….48

**Fig. 54:**
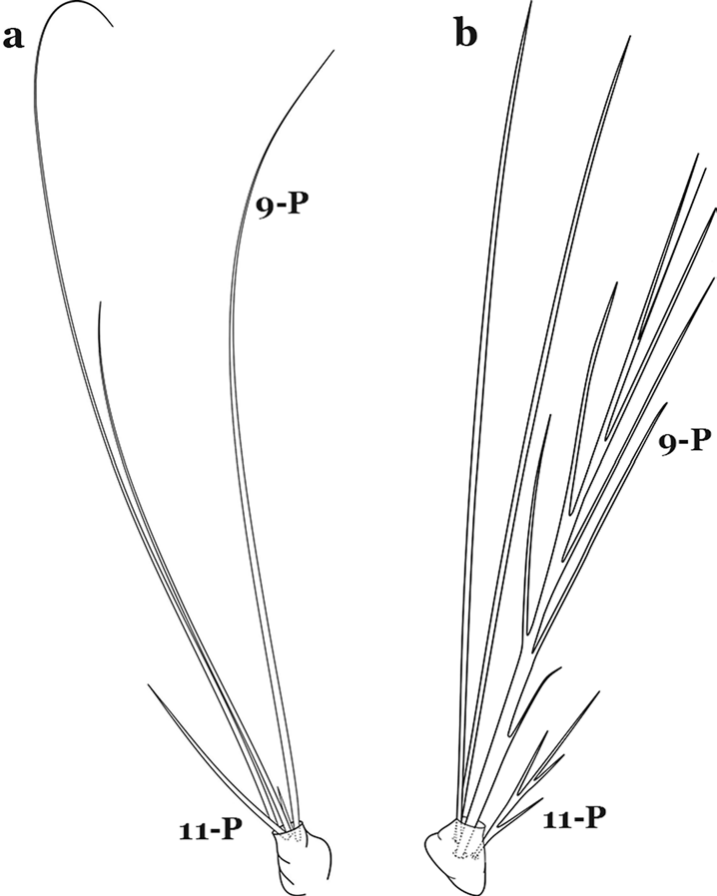
Larval prothoracic setae 9,11-P. **a** *An. mattogrossensis*. **b** *An. squamifemur* Antunes, 1937
- Seta 9-P branched (Fig. 54b)…….53
- Ventral surface of thorax and abdomen spiculose…….*An*. *minor*
- Ventral surface of thorax and abdomen smooth…….49
- Seta 3-C with 3 or more branches or dendritic (Fig. 55a)…….50

**Fig. 55:**
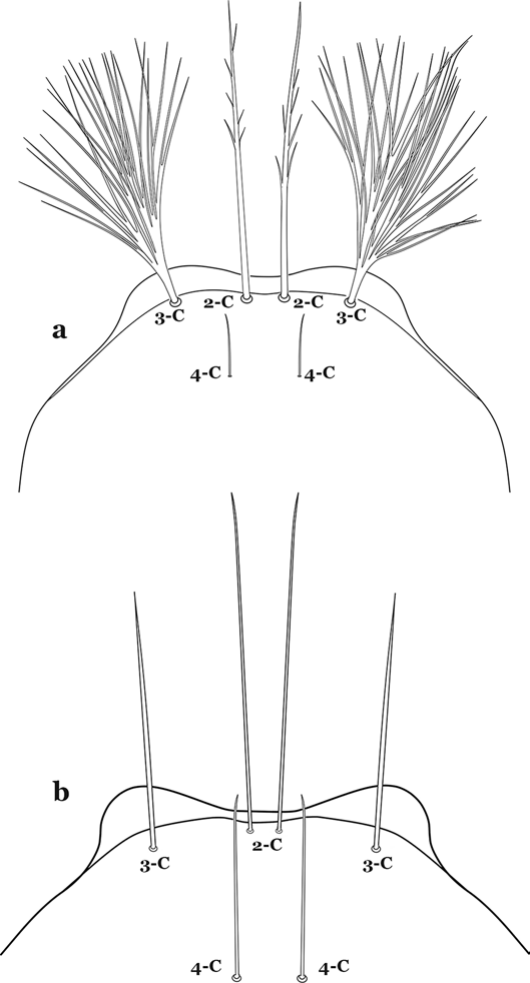
Larva head, dorsal view, setae 2–4-C. **a** *An. mattogrossensis.* **b** *An. tibiamaculatus* (Neiva, 1906)
- Seta 3-C single or double (Fig. 55b)…….52
- Seta 1-P fan-like (Fig. 56a)…….*An*. *mattogrossensis* (in part)

**Fig. 56:**
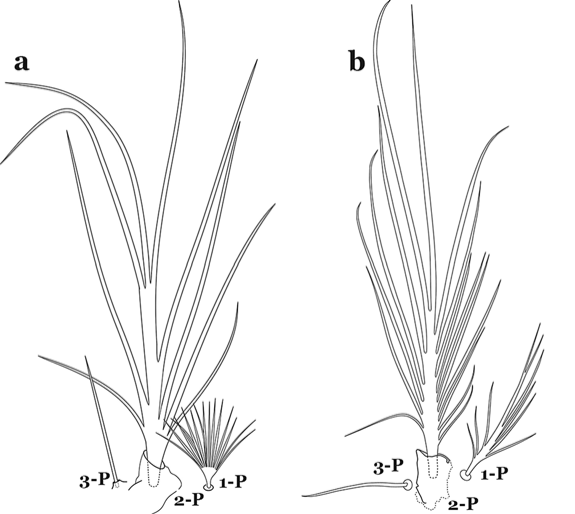
Larval prothoracic setae 1–3-P. **a** *An. mattogrossensis*. **b** *An. peryassui* Dyar & Knab, 1908
- Seta 1-P single or pinnate (Fig. 56b)…….51
- Seta 3-C strongly dendritic, branching begins near base, with more than 30 branches, shorter than 2-C; seta 4-C branching begins near base (Fig. 57a)…….*An*. *peryassui*

**Fig. 57:**
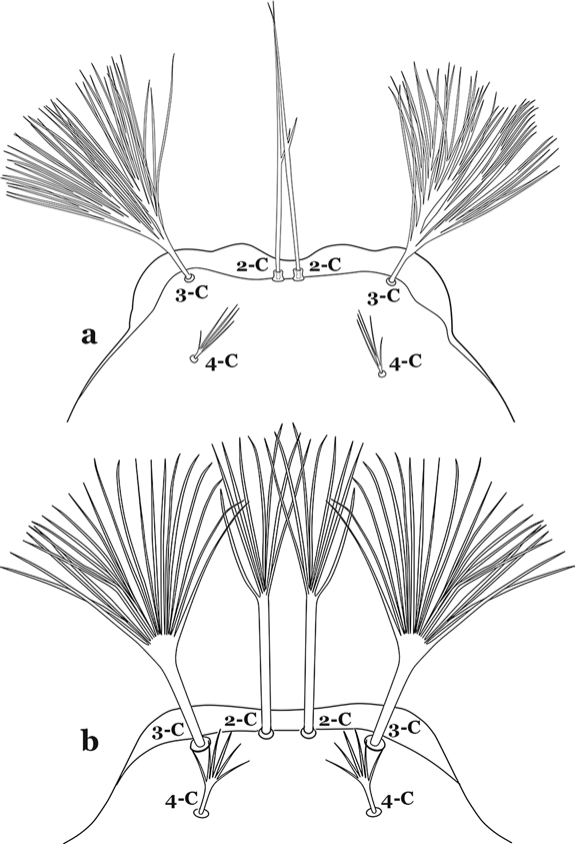
Larva head, dorsal view, setae 2–4-C. **a** *An. peryassui*. **b** *An. shannoni* Davis, 1931
- Seta 3-C branching begins near mid-length (Fig. 57b); other characters, variable…….*An*. *annulipalpis*, *An*. *shannoni* & *An*. *vestitipennis*
- Seta 3-C shorter than 0.5 length of 2-C (Fig. 58a)…….*An*. *eiseni eiseni* & *An*. *eiseni geometricus*

**Fig. 58:**
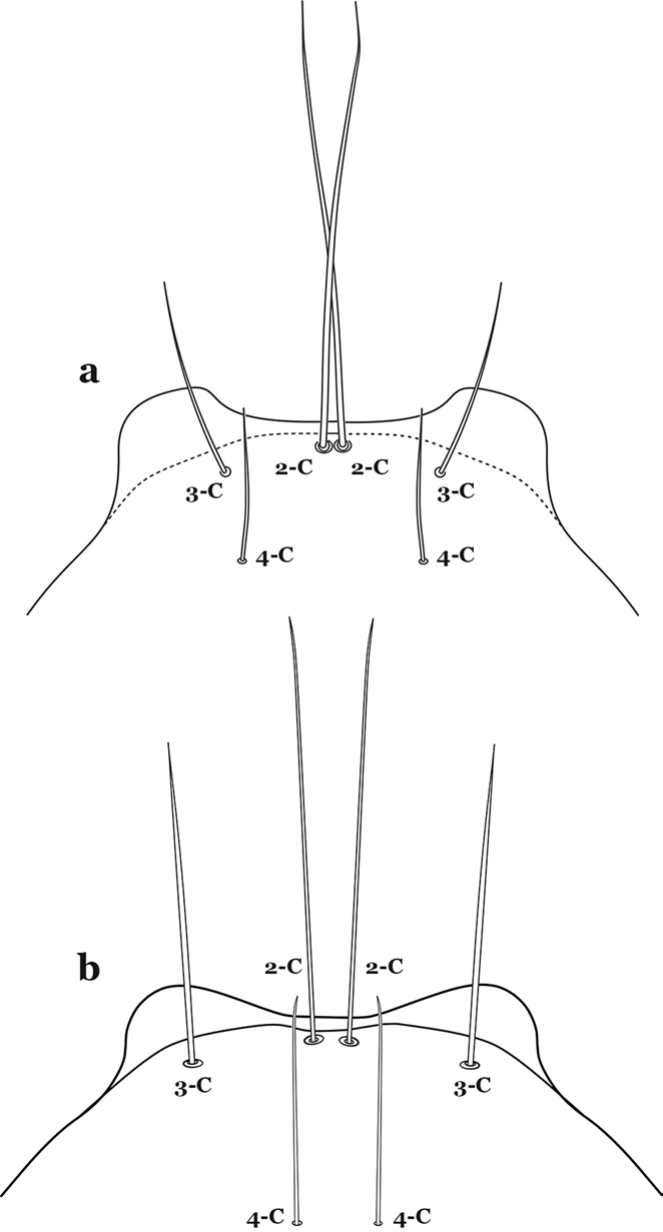
Larva head, dorsal view, setae 2–4-C. **a** *An. eiseni* Coquillett, 1902. **b** *An. tibiamaculatus* (Neiva, 1906)
- Seta 3-C about 0.67 length of 2-C (Fig. 58b)…….*An*. *tibiamaculatus*
- Seta 4-C very small or inconspicuous, 1–3-branched (Fig. 59a)…….*An*. *gilesi* & *An*. *squamifemur*

**Fig. 59:**
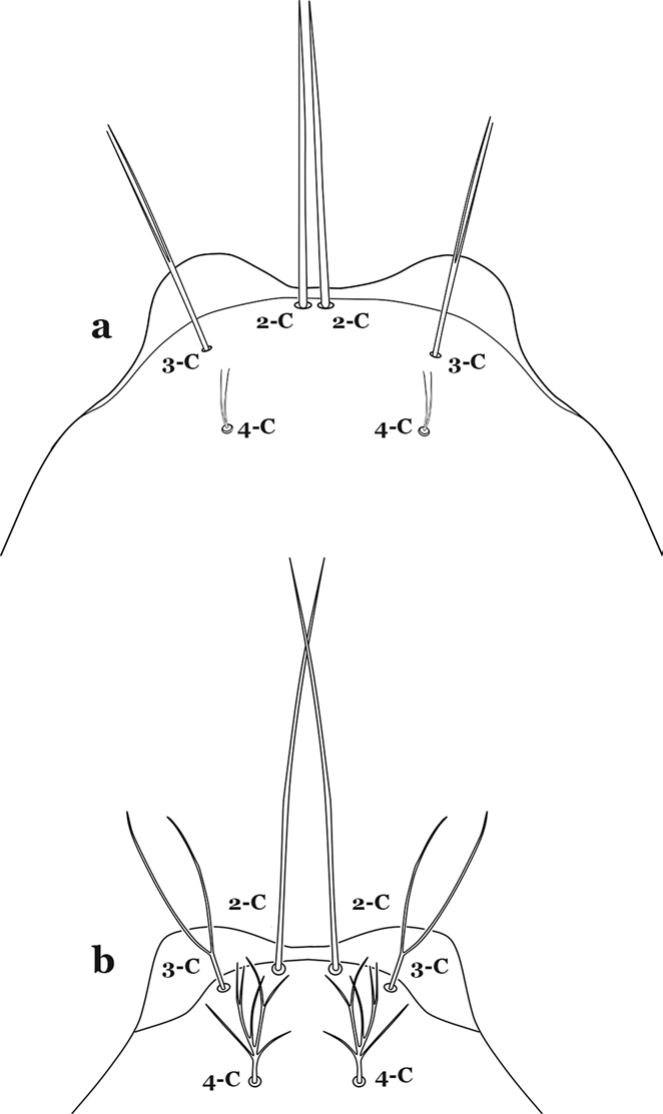
Larva head, dorsal view, setae 2–4-C. **a** *An. gilesi* Neiva, 1908. **b** *An. vargasi* Gabaldon, Cova-Garcia & Lopez, 1941
- Seta 4-C long, single or multi-branched (Fig. 59b)…….54
- Setae 3,4-C single…….*An*. *pseudotibiamaculatus*
- Seta 3-C double; seta 4-C multi-branched (Fig. 59b)…….*An*. *vargasi*
- Seta 3-C single or double (Fig. 60a)…….57

**Fig. 60:**
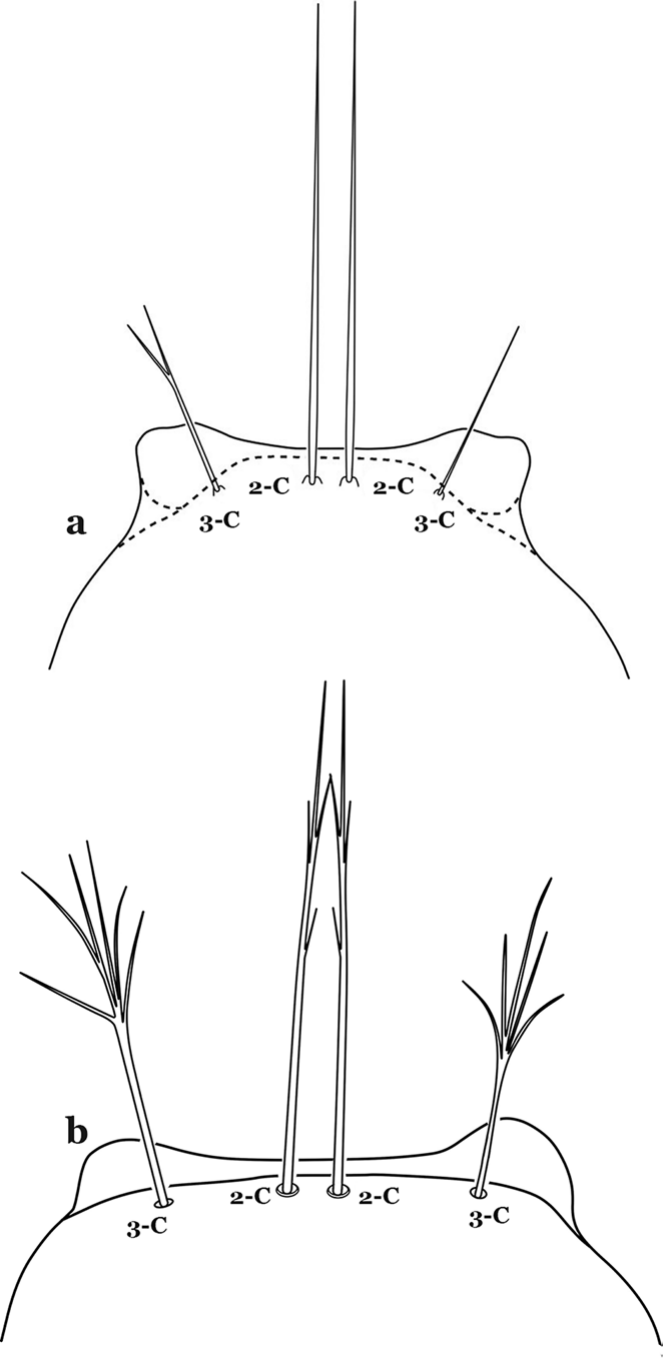
Larva head, dorsal view, setae 2,3-C. **a** *An. neomaculipalpus* Curry, 1931. **b** *An. calderoni*
- Seta 3-C multi-branched (Fig. 60b)…….58
- Seta 1-P single or with few branches (Fig. 61); setae 9–12-P single…….*An*. *neomaculipalpus*

**Fig. 61:**
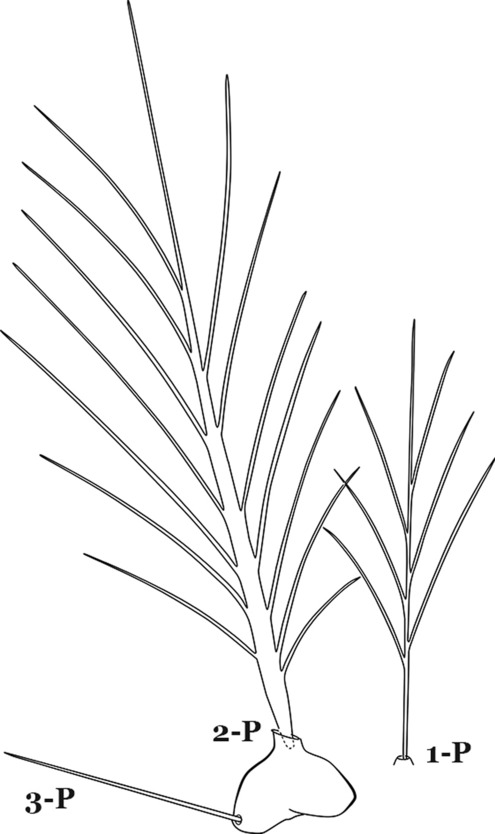
*An. neomaculipalpus*, larval prothoracic setae 1–3-P
- Seta 1-P multi-branched; seta 9-P with few lateral branches, setae 10–12-P single…….*An*. *apicimacula*
- Seta 1-P branched distally, fan-like (Fig. 62a)…….*An*. *mattogrossensis* (in part)

**Fig. 62:**
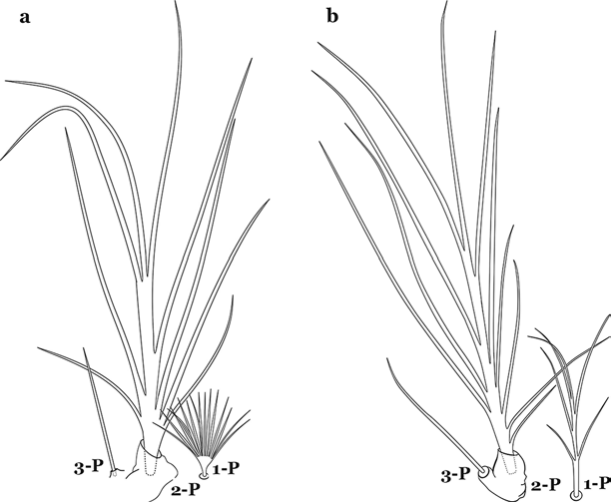
Larval prothoracic setae 1–3-P. **a** *An. mattogrossensis*. **b** *An. calderoni*
- Seta 1-P branched or pinnate (Fig. 62b)…….58
- Setae 9–12-P all single (Fig. 63a)…….59

**Fig. 63:**
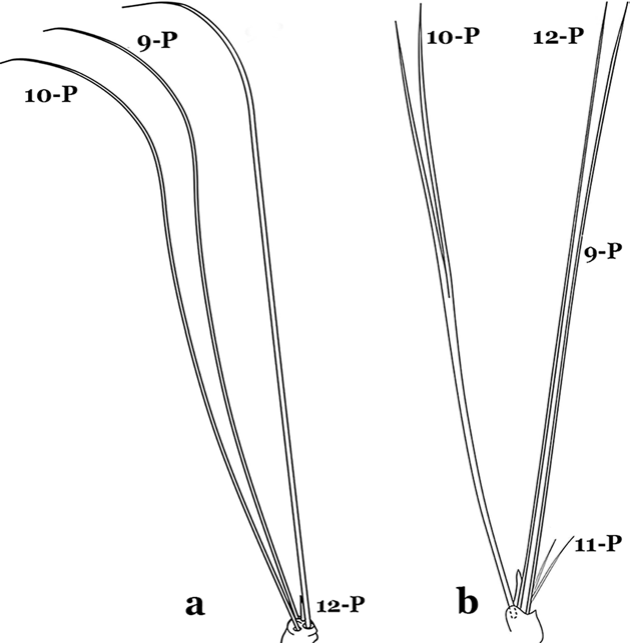
Larval prothoracic setae 9–12-P. **a** *An. anchietai* Corrêa & Ramalho, 1968. **b** *An. calderoni*
- Setae 9–12-P with at least one branched (Fig. 63b)…….61
- Seta 1-X inserted on saddle (Fig. 64a)…….*An*. *malefactor*

**Fig. 64:**
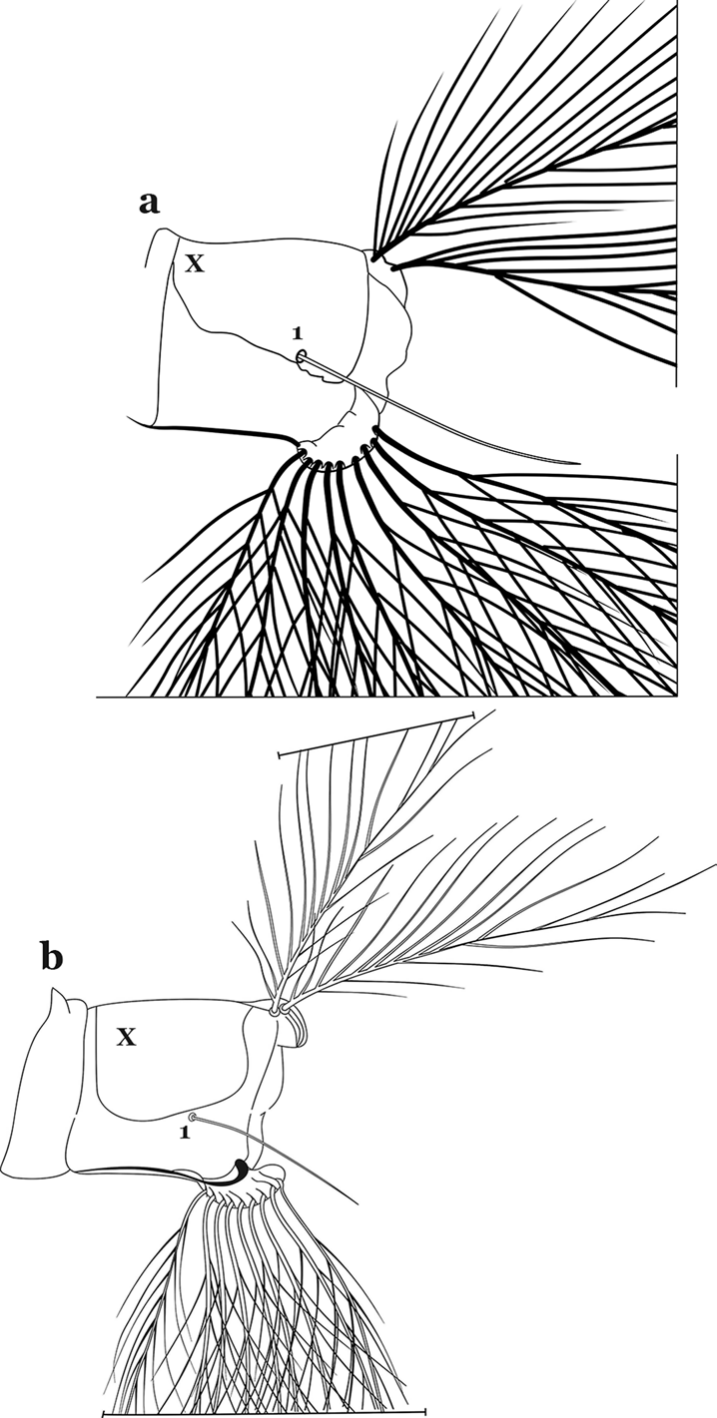
Abdominal segment X. **a** *An. malefactor* Dyar & Knab, 1907. **b** *An. mediopunctatus* (Lutz, 1903)
- Seta 1-X not inserted on saddle (Fig. 64b)…….60
- Seta 4-A long with lateral branches (Fig. 65a)…….*An*. *calderoni*, *An*. *guarao* & *An*. *punctimacula*

**Fig. 65:**
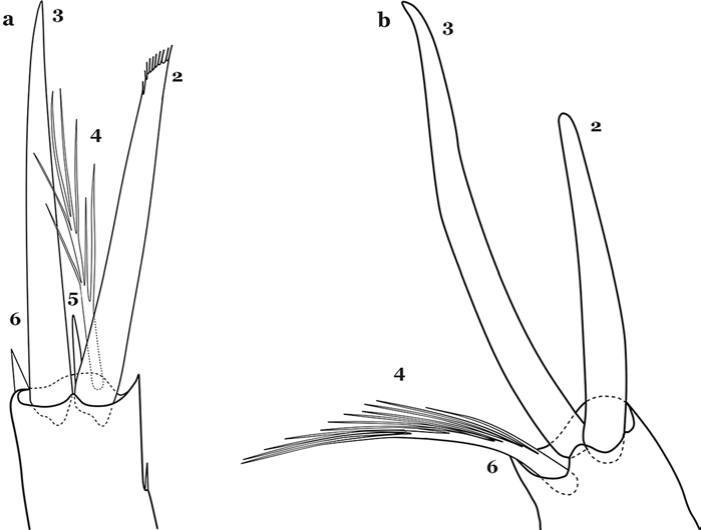
Antennal setae 2–6-A. **a** *An*. *calderoni*. **b** *An. mediopunctatus*
- Seta 4-A short and asymmetrically branched (Fig. 65b)…….*An*. *costai*, *An*. *forattinii* & *An*. *mediopunctatus*
- Seta 3-C with few short branches originating on distal half of main stem (Fig. 66a)…….*An*. *medialis*

**Fig. 66:**
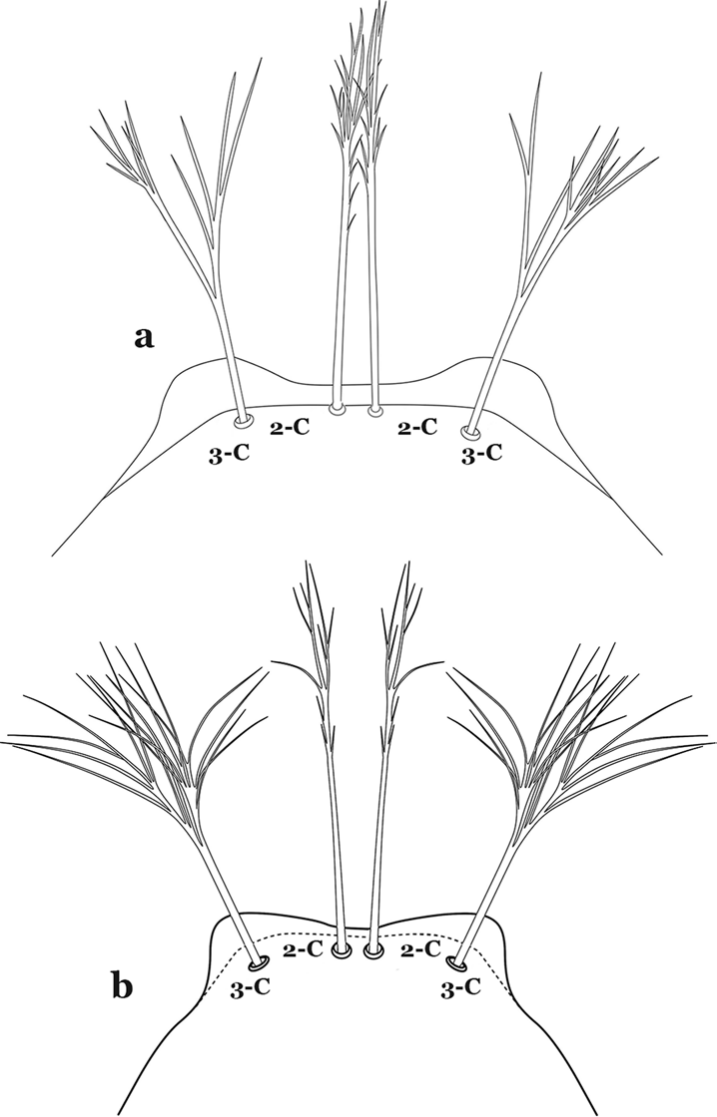
Larva head, dorsal view, setae 2,3-C. **a** *An. medialis* Harbach, 2018. **b** *An. fluminensis* Root, 1927
- Seta 3-C with long branches originating on proximal half of main stem (Fig. 66b)…….62
- Seta 1-P single or double (Fig. 67a)…….*An*. *maculipes* & *An*. *anchietai*

**Fig. 67:**
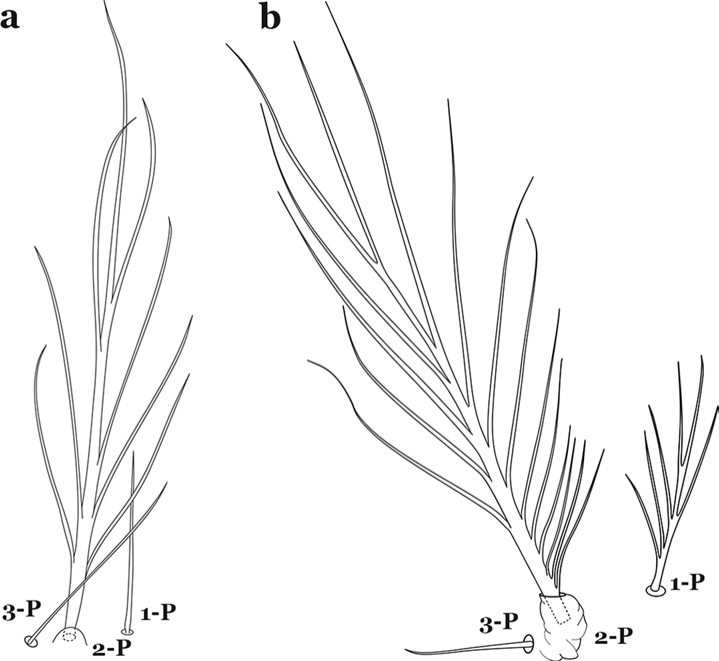
Larval prothoracic setae 1–3-P. **a** *An. anchietai*. **b** *An. fluminensis*
- Seta 1-P with multiple branches (Fig. 67b)…….*An*. *fluminensis*

## Conclusions

Traditional and new characters have been employed in the key. Characters of the spiracular apparatus were found to be useful for the identification of certain species, such as those of the Strodei Group and the Myzorhynchella Section. The character states of abdominal seta 6-IV,V being single *versus* being branched has been largely employed to separate species of the Myzorhynchella Section from those of *Nyssorhynchus*. However, this character needs to be carefully considered because of variation in species of Myzorhynchella Section. In addition, in *An. atacamensis* and *An. pictipennis* this seta is slightly serrate at the edges. The variation in the character was included in the key to avoid inaccurate identification of the species of *Nyssorhynchus* as species of the subgenus *Anopheles*.

## Data Availability

Specimens used in the current study are deposited and available in the Coleção Entomológica de Referência, Faculdade de Saúde Pública, Universidade de São Paulo (FSP-USP), São Paulo State, Brazil, the US National Mosquito Collection, Smithsonian Institution, Washington, DC, USA (USNMC), and the Facultad de Ciencias Naturales y Exactas de la Universidad del Valle, Colombia.
